# Exploring Gut Microbiota in Patients with Colorectal Disease Based on 16S rRNA Gene Amplicon and Shallow Metagenomic Sequencing

**DOI:** 10.3389/fmolb.2021.703638

**Published:** 2021-07-09

**Authors:** Yuanfeng Liu, Xiang Li, Yudie Yang, Ye Liu, Shijun Wang, Boyang Ji, Yongjun Wei

**Affiliations:** ^1^ Department of Vascular and Endovascular Surgery, First Affiliated Hospital of Zhengzhou University, Zhengzhou, Henan, China; ^2^ Science China Press, Beijing, China; ^3^ Key Laboratory of Advanced Drug Preparation Technologies, School of Pharmaceutical Sciences, Ministry of Education, Zhengzhou University, Zhengzhou, China; ^4^ Laboratory of Synthetic Biology, Zhengzhou University, Zhengzhou, China; ^5^ Oncology Department, Colorectal and Anal Surgery Department, The First Affiliated Hospital of Zhengzhou University, Zhengzhou, Henan, China; ^6^ Department of Biology and Biological Engineering, Chalmers University of Technology, Gothenburg, Sweden

**Keywords:** gut microbiota, colorectal diseases, 16S amplicon, network analysis, shallow metagenomic sequencing

## Abstract

The gastrointestinal tract, the largest human microbial reservoir, is highly dynamic. The gut microbes play essential roles in causing colorectal diseases. In the present study, we explored potential keystone taxa during the development of colorectal diseases in central China. Fecal samples of some patients were collected and were allocated to the adenoma (Group A), colorectal cancer (Group C), and hemorrhoid (Group H) groups. The 16S rRNA amplicon and shallow metagenomic sequencing (SMS) strategies were used to recover the gut microbiota. Microbial diversities obtained from 16S rRNA amplicon and SMS data were similar. Group C had the highest diversity, although no significant difference in diversity was observed among the groups. The most dominant phyla in the gut microbiota of patients with colorectal diseases were Bacteroidetes, Firmicutes, and Proteobacteria, accounting for >95% of microbes in the samples. The most abundant genera in the samples were *Bacteroides*, *Prevotella*, and *Escherichia/Shigella*, and further species-level and network analyses identified certain potential keystone taxa in each group. Some of the dominant species, such as *Prevotella copri*, *Bacteroides dorei*, and *Bacteroides vulgatus*, could be responsible for causing colorectal diseases. The SMS data recovered diverse antibiotic resistance genes of tetracycline, macrolide, and beta-lactam, which could be a result of antibiotic overuse. This study explored the gut microbiota of patients with three different types of colorectal diseases, and the microbial diversity results obtained from 16S rRNA amplicon sequencing and SMS data were found to be similar. However, the findings of this study are based on a limited sample size, which warrants further large-scale studies. The recovery of gut microbiota profiles in patients with colorectal diseases could be beneficial for future diagnosis and treatment with modulation of the gut microbiota. Moreover, SMS data can provide accurate species- and gene-level information, and it is economical. It can therefore be widely applied in future clinical metagenomic studies.

## Introduction

According to global cancer statistics 2020 by GLOBOCAN, among the 36 kinds of cancers, colorectal cancer is the third most common malignancy and the second most lethal tumor in 185 countries (Sung et al., 2021). It is associated with a high mortality rate, with 0.93 million deaths reported worldwide in 2020 (Sung et al., 2021). Research suggests that genetic factors are responsible for 12–35% of all colorectal cancers (Lichtenstein et al., 2000; Czene et al., 2002; Song et al., 2020; Rajamäki et al., 2021). The human intestine is the largest microbial reservoir. The number of gut microbes is nearly equal to that of the cells present in the entire body (Wang et al., 2017), while the number of genes from the gut microbiota is 100-fold greater than that from the human genome (Bilen et al., 2018). Intestinal microbes are associated with diverse colorectal diseases, including colorectal carcinoma (Wong and Yu, 2019; Schmitt and Greten, 2021; Xing et al., 2021).

Previous studies have reported the association of some important gut microbial species with colorectal cancer (Dai et al., 2018; Wirbel et al., 2019; Yachida et al., 2019). *Fusobacterium nucleatum* (Brennan and Garrett, 2019; Rubinstein et al., 2019), certain *Escherichia coli* strains (Wassenaar, 2018), some *Bacteroides fragilis* strains (Sears et al., 2014; Chung et al., 2018), and several other bacterial species have been linked to colorectal cancer (Dai et al., 2018; Butt et al., 2021). Some of these strains induce colorectal carcinogenesis by generating metabolites or toxins (Wong and Yu, 2019). A driver–passenger model was proposed for assessing the genetic pattern of colorectal cancer progression (Tjalsma et al., 2012). The driver microbes, including the toxigenic microbes of *E. coli* and *B. fragilis* strains, damaged the host DNA and disrupted colonic barriers (Cuevas-Ramos et al., 2010; Goodwin et al., 2011; Pleguezuelos-Manzano et al., 2020; Butt et al., 2021; Lopez et al., 2021), whereas the passenger microbes, such as *F. nucleatum*, proliferated in the suitable niche created by the driver microbes (Tjalsma et al., 2012; Shang and Liu, 2018). The dysbiosis of the micro-niche may lead to colorectal cancer (Brennan and Garrett, 2016; Tilg et al., 2018). Similarly, microbial profiling of gastric cancer samples suggested that *Helicobacter pylori* may be the driver microbe and several opportunistic pathogenic microbes may directly cause gastric cancer (Li et al., 2021).

Colorectal adenoma is the main precursor of most colorectal cancers (Corley et al., 2014; Click et al., 2018). Hemorrhoid is a common colorectal disease with symptoms different from those of colorectal adenoma and colorectal cancer (Jandhyala et al., 2015). Therefore, exploring the gut microbiota of different colorectal diseases could reveal the microbial mechanism of action in colorectal carcinogenesis. The 16S rRNA gene amplicon and metagenomic strategies are commonly used to study the complex gut microbiota involved in colorectal diseases, including colorectal cancer (Flemer et al., 2017; Saito et al., 2019). The 16S rRNA gene amplicon sequencing provides insight into the diversity of microbial communities at the genus level, whereas the metagenomic strategy provides the species- and gene-level information of the gut microbiota (Wei et al., 2020b; Liang et al., 2021). Typically, the 16S rRNA gene amplicon strategy is economical than the metagenomic strategy and the associated library construction is complex (Laudadio et al., 2018; Rausch et al., 2019; Shi et al., 2020). The shallow metagenomic sequencing (SMS) strategy has been preferred to the 16S rRNA gene amplicon strategy in certain microbiome studies (Zhu et al., 2021). The use of the SMS strategy may decrease the costs associated with metagenomic sequencing strategy (Hillmann et al., 2018; Santiago-Rodriguez et al., 2020). A comparative analysis of infant gut microbiota indicated that 16S rRNA gene amplicon and metagenomic sequencing approaches can provide similar alpha and beta diversity of the gut microbiota (Peterson et al., 2021).

In this study, we used 16S rRNA gene amplicon and SMS strategies to provide insights into the gut microbiota of patients with three colorectal diseases, namely colorectal cancer, adenoma, and hemorrhoid. We compared alpha and beta diversities between the two strategies and identified potential keystone taxa associated with colorectal diseases. Moreover, antibiotic resistant and other functional gene profiles were recovered using SMS data. We also discuss the future prospects of the use of sequencing strategies in determination of the gut microbiota in colorectal diseases.

## Materials and Methods

### Ethics Statement and Sample Collection

This study was approved by the Institutional Review Board of the First Affiliated Hospital, Zhengzhou University, Zhengzhou, Henan Province, China. All patients agreed to participate and provided written informed consents. Patient details were anonymized for all analyses.

A total of 16 fecal samples were collected from 16 hospitalized patients. The 16 patients were allocated to the Group A (adenoma), Group C (colorectal cancer), or Group H (hemorrhoids; Supplementary Table S1).

The fecal samples were collected in sterile 5-ml tubes containing 2 ml DNA/RNA shield liquids, which were provided by DeepBiome Co., Ltd. The collected samples were sent to DeepBiome Co., Ltd. for 16S rRNA gene amplicon and SMS analyses. Patient information including smoking and alcohol intake history and antibiotic use history was obtained.

### 16S rRNA Gene Amplicon Sequencing

The DNA from samples was extracted using DNeasy PowerSoil Kit (Qiagen, Germany). Approximately 500 µL of the fecal sample from each tube was used for DNA extraction. The DNA was quantified using Nanodrop 2000 (Thermo Fisher Scientific, United States), and the V3-V4 regions of the 16S rRNA gene sequences were amplified using KAPA HiFi HotStart ReadyMix PCR Kit KK2600 (Roche Sequencing, South Africa), according to the manufacturer’s instructions. The amplified PCR fragments were purified and mixed (Liang et al., 2019; Jiang et al., 2020; Mai et al., 2020). The sequencing of mixed PCR fragments was performed by DeepBiome Co., Ltd.

### 16S rRNA Amplicon Data Analyses

The raw fastq files were processed using atlas-utils, and barcode and low-quality sequences were filtered using Trimmomatic (Wei et al., 2020a). After filtering, the chimeric reads were removed using atlas-utilis. Paired-end reads were merged with default parameters by using Usearch software v.11.0.667. Finally, the sequences were classified into operational taxonomic units (OTUs), with a cutoff of 97% identity (Cole et al., 2014). The OTU tables were generated using Usearch software. The alpha parameters of the samples were generated using the Usearch alpha_div tool (Wei et al., 2020a). The beta-diversity distance and differences between the samples and the OTU table were evaluated using the Usearch beta_div tool. The unweighted pair–group method with arithmetic mean (UPGMA) was used, and principal coordinates analysis (PCoA) was performed using R software. MetagenoNets was used to infer microbial networks in the study groups (Nagpal et al., 2020). A Pearson vanilla algorithm was used for network construction. The *p* value was significant at 0.05, and the critical r-value cutoff was 0.7 (Li et al., 2021). The network figures were generated according to the instructions of MetagenoNets (Nagpal et al., 2020).

### Metagenomic Sequencing

The DNA used for the SMS was the same as that used for 16S rRNA amplicon sequencing. The DNA was quantified using Qubit dsDNA HS assay kit (12640ES60, Yeason Biotechnology, Shanghai, China). KAPA HyperPrep Kit (Illumina; KK8504), KAPA Dual-Indexed Adapters (KK8722), KAPA pure beads, and KAPA Library Quantification Kit (KK4824) were used to construct the SMS library (Liang et al., 2021). The SMS library was sequenced using the Illumina platform, and approximately 1 Gbp data were generated after sequencing.

### SMS Data Analyses

The quality of raw SMS data was evaluated using FastqQC (version 0.11.9), and adaptor sequences in raw reads were removed using Trimmomatic (version 0.38 (Bolger et al., 2014). Kraken2 was used for taxonomic classification of SMS data. The sequence reads were assembled using metaSPAdes (version 3.13.2) with the parameters of −k 21,33,55 (Nurk et al., 2017). eggnog-mapper (version 0.12.7) was used for COG term assignment, with KOfam parameters of −e 1e-3 (Huerta-Cepas et al., 2017). The gene-level TPM values of the genes were calculated (Wei et al., 2019). AMRFinder Plus (version 3.1.1b) was used to annotate the antimicrobial resistance genes in the SMS data (Feldgarden et al., 2019).

All 16S rRNA gene amplicon and SMS data were deposited in the GenBank database, with the BioProject accession number of PRJNA725613, with the BioSample accession numbers of SAMN1889570-SAMN18895718, the SRA accession numbers of 16S rRNA data are SRR14410510-SRR14410525, the SRA accession numbers of SMS data are SRR14460530-SRR14460545.

## Results

### Microbial Profiles of the Samples

The numbers of samples obtained from Groups A, C, and H were 6, 4, and 6, respectively (Supplementary Table S1). The 16S rRNA gene fragment sequencing generated 1,457,234 reads, with an average of 91,077 sequences per sample (Supplementary Table S1). The sizes of SMS data for these samples were 0.45–5.76 G (average: 1.6 G) (Supplementary Table S2).

The sequences of these samples were classified into OTUs based on 97% identity. The OTUs ranged from 105 to 314 (Table 1). The average OTUs of Groups A, C, and H were 162.17 ± 32.79, 251.75 ± 21.36, and 239.83 ± 52.05, respectively (Table 1). Group C demonstrated to have the highest diversity (Table 1), although no significant difference in diversity was observed among the groups. SMS data also demonstrated similar results, with Group C having the highest diversity (Supplementary Table S3).

**TABLE 1:** Alpha parameters of the three groups based on 16S rRNA gene amplicon data.

	Richness	Chao1	Shannon_2	Simpson	Dominance	Equitability
Group A	162.17 ± 32.79	222.95 ± 29.12	3.45 ± 0.78	0.21 ± 0.08	0.79 ± 0.08	0.47 ± 0.09
Group C	251.75 ± 21.36	291.58 ± 11.38	5.15 ± 0.50	0.09 ± 0.06	0.91 ± 0.06	0.65 ± 0.06
Group H	239.83 ± 52.05	295.7 ± 68.72	4.16 ± 0.47	0.16 ± 0.05	0.842 ± 0.05	0.53 ± 0.06

### Microbial Diversity at the Phylum and Genus Levels

At the phylum level, the most dominant phyla were Bacteroidetes, Firmicutes, and Proteobacteria (Table 2). The most dominant phylum in all the groups was Bacteroidetes, with the following compositions: 44.41 ± 19.76% in Group A, 52.95 ± 7.33% in Group C, and 59.04 ± 17.41% in Group H (Table 2). The second most dominant phylum in all the groups was Firmicutes, and its percentage in Group C was higher (40.44 ± 6.63%) than in Groups A and H. Proteobacteria was the third most dominant phylum, with a higher percentage in Group A (24.97 ± 20.75%) than in Group C (3.95 ± 1.74%) and Group H (8.28 ± 12.12%; Table 2). The percentage of *Fusobacteria* in Group A (3.16 ± 6.20%) was higher than in Group C (0.4 ± 0.77%) and Group H (0.07 ± 0.08%; Table 2). The 16S rRNA gene data revealed that the microbial distribution was different in samples from the same groups at phylum and genus levels (Supplementary Figure S1).

**TABLE 2:** Phylum distribution of the three groups based on 16S rRNA gene amplicon data.

	Group A	Group C	Group H
*Bacteroidetes*	44.41 ± 19.76%	52.95 ± 7.33%	59.04 ± 17.41%
*Firmicutes*	26.45 ± 15.22%	40.44 ± 6.63%	30.7 ± 11.69%
*Proteobacteria*	24.97 ± 20.75%	3.95 ± 1.74%	8.28 ± 12.12%
*Fusobacteria*	3.16 ± 6.20%	0.4 ± 0.77%	0.07 ± 0.08%
*Actinobacteria*	0.97 ± 1.21%	0.66 ± 0.17%	1.85 ± 1.83%
*Others*	0.01 ± 0.06%	0.32 ± 2.25%	0.01 ± 0.09%

The SMS data were similar to the 16S rRNA data, although the richness of SMS data was higher than that of 16S rRNA data (Table 1 and Supplementary Table S2). Bacteroidetes, Firmicutes, and Proteobacteria (Supplementary Table S4) were the most dominant phyla in the samples. However, their compositions in the groups were found to differ between 16S rRNA and SMS data (Table 2 and Supplementary Table S4). Importantly, the percentage of *Bacteroidota* in Group A was 49.76%, whereas it was 61.66 and 75.79% in Groups C and H, respectively (Supplementary Table S4). The percentage of *Bacteroidota* in Groups C and H based on SMS data was higher than its corresponding composition in each group based on 16S rRNA data (Table 2 and Supplementary Table S4).

Based on 16S rRNA data, the most dominant genera were assigned to *Bacteroides*, *Prevotella*, *Escherichia or Shigella*, *Faecalibacterium*, and *Blautia* (Table 3). *Bacteroides* and *Prevotella* were the most dominant genera in all the groups (Table 3). The percentages of *Bacteroides* in Groups A, C, and H were 28.88 ± 23.70%, 22.2 ± 19.66%, and 33.8 ± 25.05%, respectively (Table 3). The percentages of *Prevotella* in Groups A, C, and H were 14.41 ± 27.19%, 26.28 ± 24.78%, and 23.12 ± 28.32%, respectively (Table 3). Although the genus compositions of the groups were different, no significant difference was observed between each group. The SMS data were similar to 16S rRNA data in terms of genus compositions. According to SMS data, the most dominant genera in all the groups were *Bacteroides* and *Prevotella*, and their compositions in each group were similar (Table 3 and Supplementary Table S5). Overall, microbial compositions at the gene level were consistent between 16S rRNA and SMS data (Table 3 and Supplementary Table S5).

**TABLE 3:** Genera distribution of the three groups based on 16S rRNA gene amplicon data.

	Group A	Group C	Group H
*Bacteroides*	28.88 ± 23.70%	22.2 ± 19.66%	33.8 ± 25.05%
*Prevotella*	14.41 ± 27.19%	26.28 ± 24.78%	23.12 ± 28.32%
Above_genus	13.01 ± 20.23%	17.79 ± 10.74%	11.17 ± 4.93%
*Escherichia/Shigella*	13.86 ± 10.47%	0.84 ± 1.21%	5.85% ± 12.01
*Faecalibacterium*	0.91 ± 1.06%	5.29 ± 3.57%	4.36 ± 3.32%
*Streptococcus*	5.82 ± 11.88%	0.1 ± 0.11%	0.22 ± 0.38%
*Blautia*	1.67 ± 1.61%	1.85 ± 0.86%	2.62 ± 2.85%
*Megamonas*	0.03 ± 0.02%	4.72 ± 6.10%	2.08 ± 1.6%
*Lachnospiracea_incertae_sedis*	0.87 ± 1.10%	1.45 ± 1.31%	2.3 ± 3.01%
*Veillonella*	3.56 ± 5.90%	0.05 ± 0.05%	0.28 ± 0.46%
*Fusobacterium*	3.15 ± 6.19%	0.4 ± 0.77%	0.07 ± 0.08%
*Phascolarctobacterium*	0.93 ± 1.81%	1.4 ± 1.92%	0.99 ± 1.87%
*Roseburia*	1.06 ± 1.71%	1.2 ± 0.85%	0.86 ± 0.65%
*Bifidobacterium*	0.71 ± 0.9%	0.24 ± 0.15%	1.73 ± 1.8%
*Parabacteroides*	0.9 ± 1.30%	1.39 ± 0.97%	0.73 ± 0.64%
*Ruminococcus*	0.47 ± 1.07%	1.22 ± 1.29%	1.12 ± 1.60%
*Clostridium_XlVa*	0.76 ± 0.90%	1.84 ± 1.59%	0.42 ± 0.36%
*Clostridium_sensu_stricto*	2.22 ± 2.90%	0.12 ± 0.18%	0.07 ± 0.07%
*Oscillibacter*	0.1 ± 0.17%	1.1 ± 1.53%	1.27 ± 2.39%
*Sutterella*	0.56 ± 1.31%	0.29 ± 0.59%	0.97 ± 1.98%
*Alistipes*	0.01 ± 0.01%	1.62 ± 0.97%	0.54 ± 0.54%
*Ruminococcus2*	0.35 ± 0.47%	0.48 ± 0.63%	0.91 ± 1.23%
*Haemophilus*	1.08 ± 1.26%	0.27 ± 0.47%	0.09 ± 0.08%
*Dialister*	0.19 ± 0.44%	1.12 ± 1.83%	0.4 ± 0.57%
*Parasutterella*	0.05 ± 0.09%	0.2 ± 0.30%	0.83 ± 0.90%
*Romboutsia*	0.83 ± 1.43%	0.12 ± 0.06%	0.1 ± 0.08%
*Dorea*	0.14 ± 0.13%	0.55 ± 0.60%	0.38 ± 0.24%
*Anaerostipes*	0.21 ± 0.26%	0.2 ± 0.20%	0.45 ± 0.72%

### Dominant Species in the Samples

Nearly all the representative sequences of dominant OTUs demonstrated >97% identity with known isolates, indicating that most microbes in the samples could be cultured (Table 4) (Lagier et al., 2016; Bilen et al., 2018). The most dominant OTU, ZOTU_4, in the groups was assigned as *Prevotella copri*, which is a common gut microbe. *P*. *copri* is associated with host health, and its diversity can be affected by the diet (De Filippis et al., 2019). The microbe has strong carbohydrate metabolism ability; however, its composition in the western populations is underrepresented (Tett et al., 2019). In our study, all the samples were from patients of central China. The high-level compositions of *P*. *copri* were in accordance with the vegetarian habit in this region. Other dominant OTUs were assigned to the *Bacteroides* or *Escherichia* strains (Table 4). Two OTUs, namely ZOTU_8 and ZOTU_5, were assigned to the pathogenic microbes, and their percentages in Group A were high. The ZOTU_8 composition in sample A5 of Group A was 43.72% and the ZOTU_5 composition in sample A3 of Group A was 29.76%. These patients (A3 and A5) had symptoms of inflammation, and it was presumed that strains representing ZOTU_8 and ZOTU_5 caused these symptoms (Table 4). In addition, ZOTU_6, ZOTU_10, and ZOTU_17 percentages in sample A6 of Group A were 29.29, 17.44, and 14.95%, respectively, which were higher than the corresponding OTU percentages of other samples in Group A (Table 4). These three dominant OTUs in sample A6 represented potential opportunistic pathogenic bacteria, which were possibly responsible for the serious adenoma symptoms (Table 4).

**TABLE 4:** OTU distribution of the three groups based on 16S rRNA gene amplicon data.

	Group A	Group C	Group H	Isolated microbes (accession numbers)	Identity (%)
ZOTU_4	4.46 ± 10.67%	17.97 ± 16.47%	17.68 ± 20.21%	*Prevotella copri* strain Pc (MT152634.1)	100.00
ZOTU_1	13.86 ± 10.47%	0.84 ± 1.21%	5.85 ± 12.01%	*Escherichia coli* strain LWY6 (CP072204.1)	100.00
ZOTU_3	6.52 ± 11.03%	3.44 ± 5.56%	6.06 ± 9.91%	*Bacteroides vulgatus* strain ADE11 (MT268992.1)	100.00
ZOTU_2	8.42 ± 20.27%	2.6 ± 3%	4.23 ± 6.37%	*Bacteroides coprocola* (NR_041278.1)	100.00
ZOTU_6	5.7 ± 11.66%	2.37 ± 4.27%	6.45 ± 12.83%	*Bacteroides dorei* strain 8,642 (MT464394.1)	100.00
ZOTU_7	0.15 ± 0.28%	6.98 ± 9.16%	8.04 ± 13.95%	*Bacteroides plebeius* DSM 17135 (NR_041277.1)	100.00
ZOTU_9	6.92 ± 16.84%	1.36 ± 2.54%	3.08 ± 7.3%	*Prevotella copri* strain DSM 108494 (MN537545.1)	100.00
ZOTU_8	7.41 ± 17.79%	0.04 ± 0.04%	0.1 ± 0.1%	*Klebsiella pneumoniae* subsp. Pneumoniae strain S1 (MW815592.1)	100.00
ZOTU_5	5.11 ± 12.08%	0 ± 0.01%	0.16 ± 0.37%	*Streptococcus pasteurianus* strain 2,323 (MT604782.1)	100.00
ZOTU_13	0.03 ± 0.02%	4.72 ± 6.1%	2.08 ± 1.6%	*Megamonas funiformis* strain JCM 14723 (CP048627.1)	100.00
ZOTU_39	0.09 ± 0.2%	3.82 ± 6.45%	1.93 ± 4.58%	*Prevotella copri* DSM 18205 (NR_113411.1)	100.00
ZOTU_10	3.07 ± 7.04%	0.58 ± 1.12%	0.37 ± 0.63%	*Bacteroides fragilis* strain ADE7 (MT268985.1)	100.00
ZOTU_21	0.1 ± 0.11%	1.09 ± 0.77%	2.9 ± 3.69%	*Merdimonas faecis* strain BR31 (NR_157642.1)	95.70
ZOTU_30	0.46 ± 0.84%	1.96 ± 2.7%	1.76 ± 2.44%	*Faecalibacterium prausnitzii* A2-165 strain JCM 31915 (CP048437.1)	100.00
ZOTU_18	0.93 ± 0.93%	0.64 ± 0.42%	2.01 ± 2.89%	Lachnospiraceae *bacterium* strain AGP2-03-00-02 (MH699332.1)	100.00
ZOTU_24	0.51 ± 1.04%	1.13 ± 1.62%	1.54 ± 2.21%	*Lactobacillus rogosae* strain ATCC 27753 (NR_104836.1)	100.00

Compared with 16S rRNA gene amplicon data, SMS data can provide species-level information. Based on SMS data, the most dominant species were assigned to *P*. *copri*, *B. dorei*, *B. vulgatus*, and few other *Bacteroides* genera (Supplementary Table S6). The percentages of *Streptococcus pasteurianus* and *Klebsiella quasipneumoniae* in samples A3 and A5 were 18.48 and 29.17%, respectively, which were similar to those obtained with 16S rRNA data. Some *B. fragilis* strains could serve as driver species in colorectal cancer (Haghi et al., 2019; Wong and Yu, 2019; Butt et al., 2021). The percentages of *B. fragilis* in Groups A, C, and H were 3.96, 0.17, and 0.43%, respectively. The percentages of another potential colorectal cancer driver species of *E. coli* in Groups A, C, and H were 2.83, 0.02, and 0.11%, respectively (Supplementary Table S6). The *F. nucleatum* percentage was not high (<0.01%) in the groups (Shang and Liu, 2018; Brennan and Garrett, 2019), suggesting the presence of other passenger opportunistic species in patients with colorectal cancer (Wong and Yu, 2019). The microbial compositions in 16S rRNA and SMS data were different, although the microbial structures were similar (Table 4 and Supplementary Table S6). Most microbes could be assigned at the species level, suggesting the possibility of isolating the human gut bacteria (Lagier et al., 2016; Bilen et al., 2018).

### Group Analysis of the Samples Based on 16S rRNA Data

**FIGURE 1:**
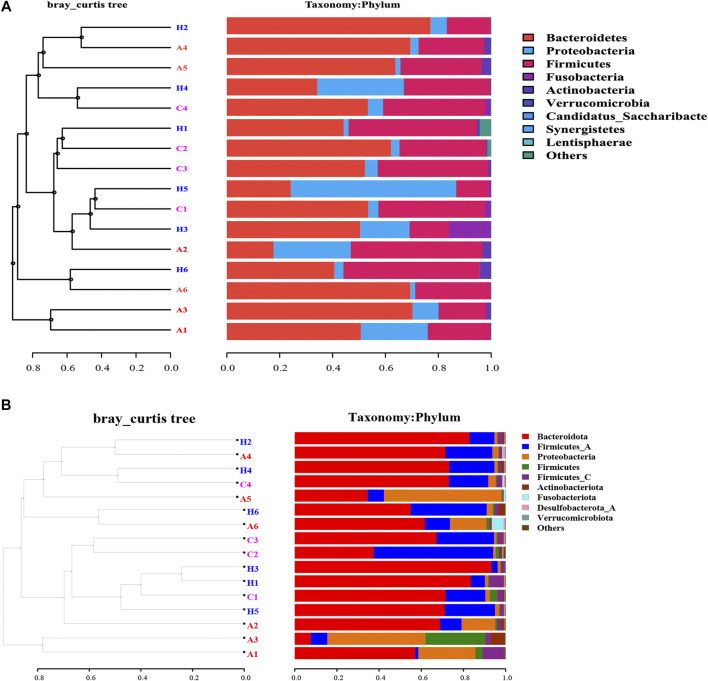
UPGMA phylogenetic tree based on **(A)** 16S rRNA data and **(B)** SMS data.

The beta-diversity analyses based on both 16S rRNA and SMS data revealed that samples from Groups A, C, and H overlapped with each other, with no significant difference (Figure 1). The UPGMA analysis based on 16S rRNA data demonstrated that samples from the same groups had different microbial profiles (Figure 1A). Samples H2, A4, A5, H4, and C4 were clustered, and so were samples H1, C2, and C3. Samples A1 and A3 and samples H6 and A6 were clustered (Figure 1A). The SMS data indicated the same results (Figure 1B). Samples A1 and A3 were clustered, and so were samples H2, A4, H4, and C4 (Figure 1B). The 16S rRNA data and the SMS data were similar (Figure 1). The phylum level distribution of these samples used for the UPGMA analysis further suggests that the 16S rRNA data were similar to the SMS data (Figure 1). The PCoA analysis was consistent with the UPGMA analysis (Figure 1 and Supplementary Figure S2).

**FIGURE 2:**
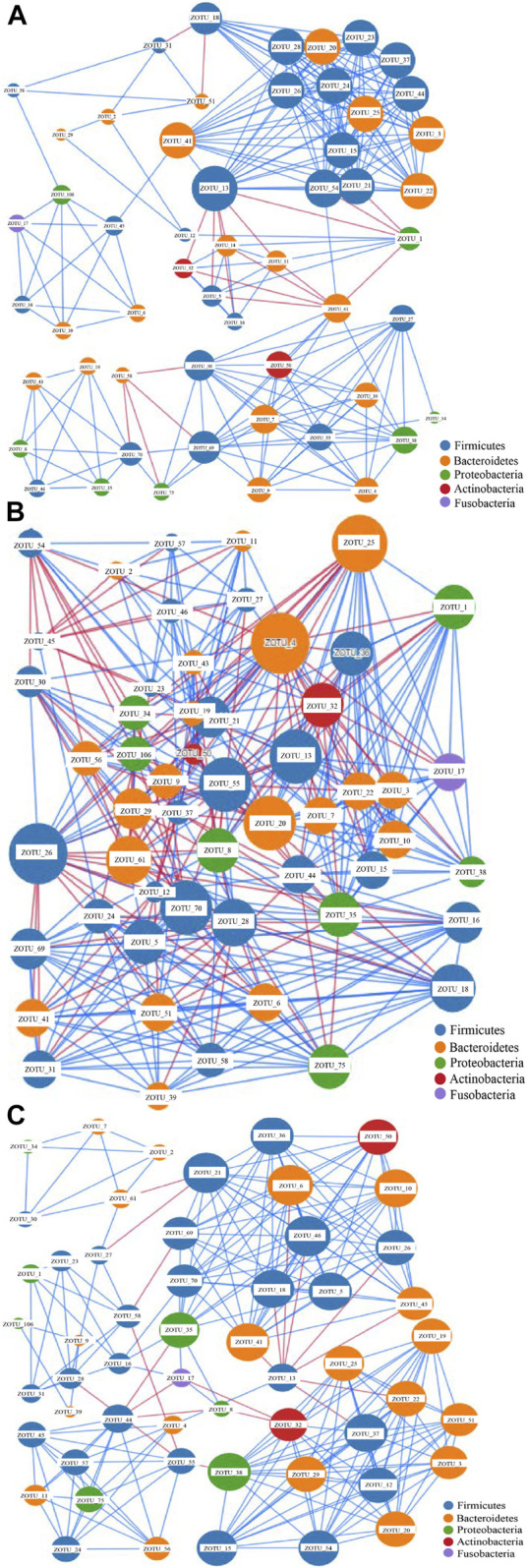
Network analysis of the gut microbiota of the three groups based on correlation analysis. **(A)** the network analysis of group A; **(B)** The network analysis of Group C; **(C)**. The network analysis of Group H.The size of each node is proportional to the node degree. The lines between two nodes colored in blue indicate positive correlation, whereas the lines between two nodes colored in blue indicate negative correlation.

The network analysis can indicate potential microbes associated with the disease, and keystone taxa can be predicted based on the microbiota data (Li et al., 2021). The most dominant 54 OTUs with a composition of >0.5% in the samples were selected for network construction. Three networks were generated (Figure 2). The edges for Groups A, C, and H were 247, 401, and 241, respectively (Figure 2 and Supplementary Table S7), and the diameters of the three groups were different. The densities of Groups A and Group H were similar, whereas that of Group C was higher than those of other groups. The average network degrees of Groups A and H were nearly 9, and that of Group C was 14.6 (Supplementary Table S7). These data revealed that the association in Group C was more complex than that in the other groups. These findings suggest the complex microbiota in patients with colorectal cancer and the highly connected correlation between the species of the microbiota (Figure 2).

The nodes in all these networks were assigned to Firmicutes, Bacteroidetes, Proteobacteria, Actinobacteria, and Fusobacteria. In Group A, the most dominant OTUs demonstrated positive correlations with other OTUs. Only four OTUs, namely ZOTU_13, ZOTU_1, ZOTU_61, and ZOTU_58, demonstrated a negative correlation with other OTUs (Figure 2A). These four OTUs may represent pathogenic species and be associated with adenoma in these patients. The OTUs ZOTU_3, ZOTU_15, ZOTU_24, and ZOTU_25 had more positive correlations than other OTUs, suggesting their importance in these samples. In Group C, both positive and negative correlations were present, and the correlations between different species were complex (Figure 2B). The keystone taxa in Group C could be ZOTU_4, ZOTU_20, ZOTU_7, ZOTU_13, and other OTUs. These OTUs are not potential pathogens, but some of them may serve as passenger microbes. Similar to Group A, the main correlations in Group H were positive (Figure 2C). Only ZOTU_13, ZOTU_44, and ZOTU_21 had negative correlations with other species (Figure 2C). Compared with the network of Group C, that of Group H was simple. The predicted keystone taxa of Group H were not pathogens, and most of them were normal human gut microbes (Figure 2C).

### Functional Profiles of the Microbiota

**FIGURE 3:**
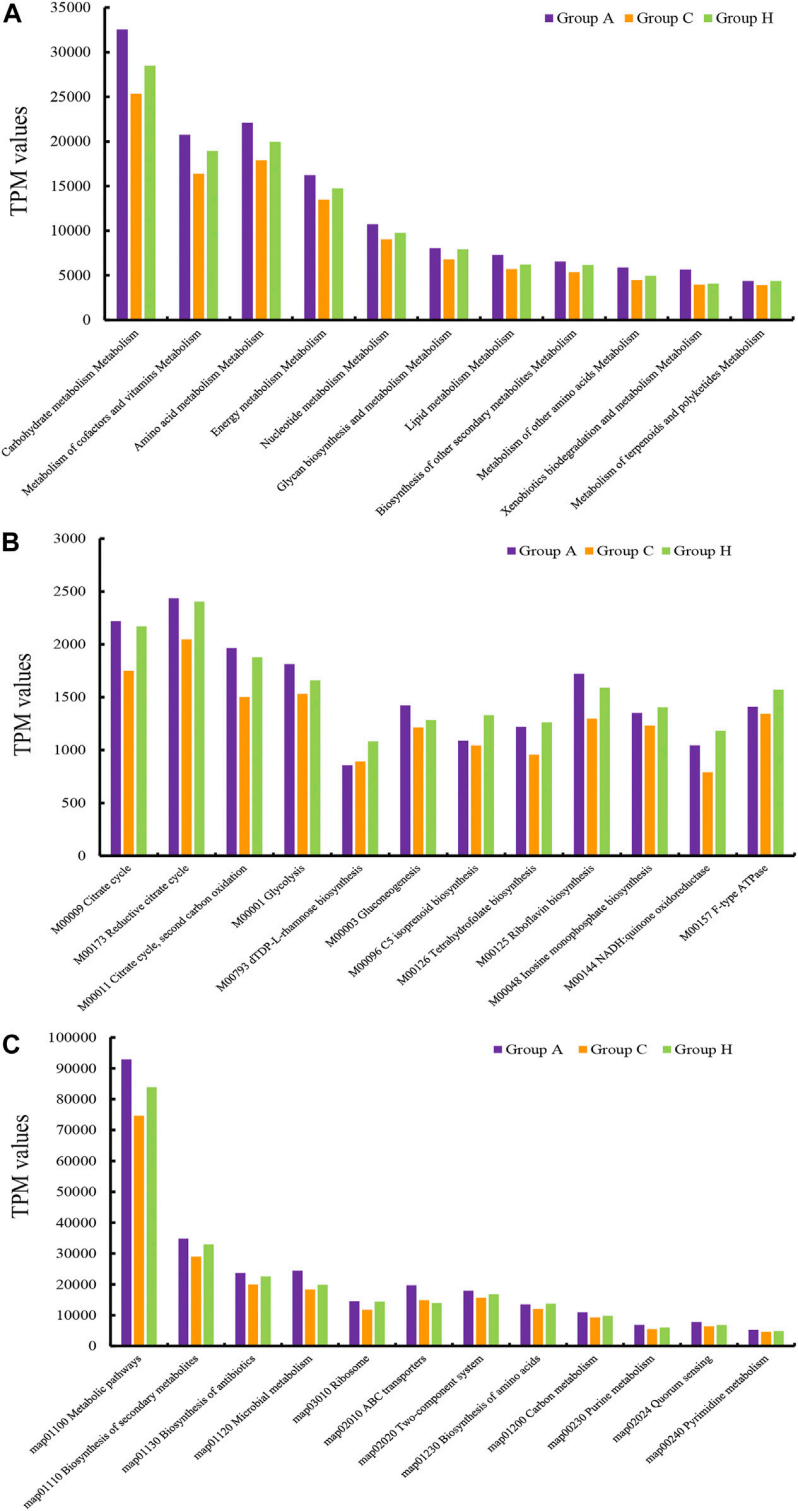
TPM values of the annotated ORFs at the **(A)** catalog level, **(B)** module level, and **(C)** pathway level.

**FIGURE 4:**
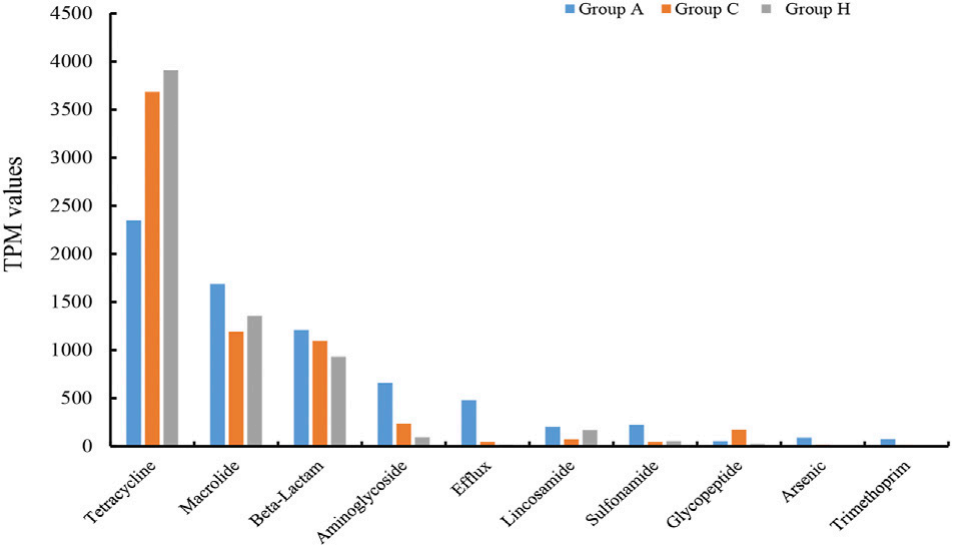
Comparison of 10 main antibiotic resistant genes in the three groups.

The assembly of SMS data generated large amounts of contigs. The assembly quality was related to the generated data. The contig N50 of all the samples ranged from 2,352 to 9,593 bp (Supplementary Table S8). The largest contigs were as high as 64.33 kbp (Supplementary Table S8). The open reading frame (ORF) in the contigs was annotated using the KEGG database. At the catalog level, the TPM values of Groups A and H were higher than that of Group C (Figure 3A). Specifically, the carbohydrate metabolism and other energy and nutrient uptake metabolism are important for microbial growth, suggesting the possible dysfunctional microbiota of Group C (Figure 3A). At the module and pathway levels, the distribution of Group C was lower than that of other two groups (Figures 3B,C). The low distribution of these metabolic catalogs in Group C suggests that the metabolic ability of gut microbiota in Group C was weak, and the microbiota of patients in Group C could be imbalanced (Figure 3B) (Gagnière et al., 2016). Some antimicrobial resistance genes were identified in some samples, and these included the antibiotic resistance genes for tetracycline, macrolide, beta-lactam, and aminoglycoside (Figure 4) (Feldgarden et al., 2019). The TPM value of tetracycline resistance gene in Group H was higher than the corresponding values in Groups A and C. For other antibiotic resistance genes, most TPM values of Group A were higher than the corresponding values of Groups C and H (Figure 4). The antibiotics tetracycline, macrolide, and beta-lactam are commonly used in clinical settings; however, their overuse could result in the high availability of corresponding antibiotic resistance genes in such samples.

## Discussion

Colorectal cancer is the third most common cancer and the second leading cause of cancer-related mortality, which suggests the importance of investigating the molecular mechanisms of colorectal carcinogenesis (Sung et al., 2021). Environmental factors, particularly gut microbes, play pivotal roles in causing colorectal diseases (Czene et al., 2002; Song and Chan, 2019). Core microbiota has been found in the gut microbiota of patients with colorectal cancer, and a study recovered the microbiota of patients with adenoma (Click et al., 2018; Saito et al., 2019). Colorectal cancer typically develops from adenoma, whereas hemorrhoids are a common colorectal disease. Comparison of these colorectal diseases may provide insights into the causative factors for colorectal carcinogenesis (Wong and Yu, 2019). In this study, we used 16S rRNA gene amplicon and SMS strategies to investigate the gut microbiota of patients with colorectal cancer, adenoma, and hemorrhoids.

Microbial profiling revealed higher diversity in Group C than in other groups, which is consistent with previous results that indicated higher microbiota diversity in patients with colorectal cancer than in healthy individuals (Tilg et al., 2018; Reis et al., 2019). The difference in microbiota diversity could be attributed to the increase in microbial diversity due to dysbiosis of gut microbiota of patients with colorectal cancer (Gao et al., 2015). The richness based on SMS data was higher than that based on 16S rRNA gene amplicon data. Metagenomic data can provide species-level microbial information, but it can lead to the overestimation of microbial richness (Wang et al., 2015). Some driver and passenger microbes were identified in the samples, but the percentages of certain known passenger species, such as *F. nucleatum*, were low (Tjalsma et al., 2012; Wong and Yu, 2019). Different ethnic groups of patients living in different areas may have diverse gut microbiota (Dai et al., 2018; Deschasaux et al., 2018; Verhaar et al., 2020). Therefore, it is possible that unknown passenger species may be present in patients of central China with colorectal disease. Although certain potential species were identified according to the network analysis, further studies based on pure cultures isolated from the gut microbiota are required to investigate their functional roles (Lagier et al., 2016; Bilen et al., 2018). In future, verification of the keystone taxa in colorectal carcinogenesis could help in identifying biomarkers for the diagnosis and prevention of colorectal diseases, including colorectal cancer (Lagier et al., 2016; Bilen et al., 2018). Moreover, modulation of the colorectal disease microbiota with probiotics or other strategies will confer health benefits to humans, which will be a value addition to the treatment paradigm (Durack and Lynch, 2019; Peng et al., 2020).

The gut microbiota is affected by diet and other environmental factors (Grazioso et al., 2019; Illiano et al., 2020). The functional profiles of the gut microbiota in the groups were different, and the metabolic catalogs of Group C were lower than those of Groups A and H. This finding indicates that the high microbial diversity of Group C could have caused dysbiosis of the microbial metabolism (Gagnière et al., 2016; Song and Chan, 2019). The identification of certain pathogenic microbes, such as *K. pneumoniae* and *S. pasteurianus*, in the samples revealed the potential of microbial analyses to recognize infective microbes in patients (Gu et al., 2021). Diverse antibiotic resistance genes were identified in SMS data. This could be due to the fact that antibiotics were administered to the hospitalized patients (Bullman et al., 2017; Cao et al., 2018). *Prevotella* and other gut microbes can be identified in oral microbiota (Yamashita and Takeshita, 2017; Qiao et al., 2020), and the oral microbiota of patients with colorectal diseases are different from that of healthy individuals (Russo et al., 2017; Flemer et al., 2018). Therefore, it is possible to classify or predict potential patients with colorectal diseases based on oral microbiota (Yamashita and Takeshita, 2017; Flemer et al., 2018). Moreover, diets can reshape the gut microbiota, implying that designing and implementing an optimized diet by using active components of plants can be an effective therapeutic strategy for colorectal diseases in the near future (Guan et al., 2020; Wang et al., 2020).

The metagenomic strategy had been widely used to identify keystone taxa in human microbiome (Saito et al., 2019). The metagenomic strategy is rapid and accurate, and could provide species- and gene-level information of the microbiota (Wang et al., 2015). However, the associated cost is high, and analysis of these data requires investment of massive computer resources (Zhu et al., 2021). The SMS approach is an affordable and rapid microbial detection strategy. Although phylum- and genus-level data are slightly different, SMS and 16S rRNA amplicon data provided similar microbial structures in this study. In addition, SMS data shed light into the species- and gene-level information, which can drive future identification of reliable microbial or gene biomarkers for diagnosis and treatment of colorectal diseases (Hillmann et al., 2018; Zhu et al., 2021).

In this study, we recovered gut microbiota from patients with three different colorectal diseases and identified the potential keystone taxa. In addition to pathogenic microbes in a few adenoma disease samples, certain passenger microbes could have roles in causing colorectal diseases. The 16S rRNA gene amplicon and SMS data provide similar alpha- and beta-diversity results, and SMS data may be applied for future clinical use in colorectal diseases. In future, designing an appropriate diet to modulate the gut microbiota may be beneficial for the prevention or treatment of colorectal diseases.

## Data Availability

The original contributions presented in the study are publicly available. This data can be found here: https://www.ncbi.nlm.nih.gov/bioproject/PRJNA725613.

## References

[B1] Bilen M. Dufour J.-C. Lagier J.-C. Cadoret F. Daoud Z. Dubourg G. (2018). The Contribution of Culturomics to the Repertoire of Isolated Human Bacterial and Archaeal Species. Microbiome 6, 94. 10.1186/s40168-018-0485-5 29793532 PMC5966928

[B2] Bolger A. M. Lohse M. Usadel B. (2014). Trimmomatic: a Flexible Trimmer for Illumina Sequence Data. Bioinformatics 30, 2114–2120. 10.1093/bioinformatics/btu170 24695404 PMC4103590

[B3] Brennan C. A. Garrett W. S. (2019). Fusobacterium Nucleatum - Symbiont, Opportunist and Oncobacterium. Nat. Rev. Microbiol. 17, 156–166. 10.1038/s41579-018-0129-6 30546113 PMC6589823

[B4] Brennan C. A. Garrett W. S. (2016). Gut Microbiota, Inflammation, and Colorectal Cancer. Annu. Rev. Microbiol. 70, 395–411. 10.1146/annurev-micro-102215-095513 27607555 PMC5541233

[B5] Bullman S. Pedamallu C. S. Sicinska E. Clancy T. E. Zhang X. Cai D. (2017). Analysis ofFusobacteriumpersistence and Antibiotic Response in Colorectal Cancer. Science 358, 1443–1448. 10.1126/science.aal5240 29170280 PMC5823247

[B6] Butt J. Jenab M. Werner J. Fedirko V. Weiderpass E. Dahm C. C. (2021). Association of Pre-diagnostic Antibody Responses to Escherichia coli and Bacteroides Fragilis Toxin Proteins with Colorectal Cancer in a European Cohort. Gut Microbes 13, 1–14. 10.1080/19490976.2021.1903825 PMC8078709 33874856

[B7] Cao Y. Wu K. Mehta R. Drew D. A. Song M. Lochhead P. (2018). Long-term Use of Antibiotics and Risk of Colorectal Adenoma. Gut 67, 672–678. 10.1136/gutjnl-2016-313413 28377387 PMC5628103

[B8] Chung L. Thiele Orberg E. Geis A. L. Chan J. L. Fu K. Destefano Shields C. E. (2018). Bacteroides Fragilis Toxin Coordinates a Pro-carcinogenic Inflammatory Cascade via Targeting of Colonic Epithelial Cells. Cell Host Microbe. 23, 203–214. 10.1016/j.chom.2018.01.007 29398651 PMC5954996

[B9] Click B. Pinsky P. F. Hickey T. Doroudi M. Schoen R. E. (2018). Association of Colonoscopy Adenoma Findings With Long-Term Colorectal Cancer Incidence. Jama 319, 2021–2031. 10.1001/jama.2018.5809 29800214 PMC6583246

[B10] Cole J. R. Wang Q. Fish J. A. Chai B. Mcgarrell D. M. Sun Y. (2014). Ribosomal Database Project: Data and Tools for High Throughput rRNA Analysis. Nucl. Acids Res. 42, D633–D642. 10.1093/nar/gkt1244 24288368 PMC3965039

[B11] Corley D. A. Jensen C. D. Marks A. R. Zhao W. K. Lee J. K. Doubeni C. A. (2014). Adenoma Detection Rate and Risk of Colorectal Cancer and Death. N. Engl. J. Med. 370, 1298–1306. 10.1056/nejmoa1309086 24693890 PMC4036494

[B12] Cuevas-Ramos G. Petit C. R. Marcq I. Boury M. Oswald E. Nougayrède J.-P. (2010). Escherichia coli Induces DNA Damage *In Vivo* and Triggers Genomic Instability in Mammalian Cells. Proc. Natl. Acad. Sci. 107, 11537–11542. 10.1073/pnas.1001261107 20534522 PMC2895108

[B13] Czene K. Lichtenstein P. Hemminki K. (2002). Environmental and Heritable Causes of Cancer Among 9.6 Million Individuals in the Swedish Family-Cancer Database. Int. J. Cancer 99, 260–266. 10.1002/ijc.10332 11979442

[B14] Dai Z. Coker O. O. Nakatsu G. Wu W. K. K. Zhao L. Chen Z. (2018). Multi-cohort Analysis of Colorectal Cancer Metagenome Identified Altered Bacteria across Populations and Universal Bacterial Markers. Microbiome 6, 70. 10.1186/s40168-018-0451-2 29642940 PMC5896039

[B15] De Filippis F. Pasolli E. Tett A. Tarallo S. Naccarati A. De Angelis M. (2019). Distinct Genetic and Functional Traits of Human Intestinal Prevotella Copri Strains Are Associated with Different Habitual Diets. Cell Host Microbe 25, 444–453. 10.1016/j.chom.2019.01.004 30799264

[B16] Deschasaux M. Bouter K. E. Prodan A. Levin E. Groen A. K. Herrema H. (2018). Depicting the Composition of Gut Microbiota in a Population with Varied Ethnic Origins but Shared Geography. Nat. Med. 24, 1526–1531. 10.1038/s41591-018-0160-1 30150717

[B17] Durack J. Lynch S. V. (2019). The Gut Microbiome: Relationships with Disease and Opportunities for Therapy. J. Exp. Med. 216, 20–40. 10.1084/jem.20180448 30322864 PMC6314516

[B18] Feldgarden M. Brover V. Haft D. H. Prasad A. B. Slotta D. J. Tolstoy I. (2019). Validating the AMRFinder Tool and Resistance Gene Database by Using Antimicrobial Resistance Genotype-Phenotype Correlations in a Collection of Isolates. Antimicrob. Agents Chemother. 63. 10.1128/AAC.00483-19 PMC6811410 31427293

[B19] Flemer B. Lynch D. B. Brown J. M. R. Jeffery I. B. Ryan F. J. Claesson M. J. (2017). Tumour-associated and Non-tumour-associated Microbiota in Colorectal Cancer. Gut 66, 633–643. 10.1136/gutjnl-2015-309595 26992426 PMC5529966

[B20] Flemer B. Warren R. D. Barrett M. P. Cisek K. Das A. Jeffery I. B. (2018). The Oral Microbiota in Colorectal Cancer Is Distinctive and Predictive. Gut 67, 1454–1463. 10.1136/gutjnl-2017-314814 28988196 PMC6204958

[B21] Gagnière J. Raisch J. Veziant J. Barnich N. Bonnet R. Buc E. (2016). Gut Microbiota Imbalance and Colorectal Cancer. World J. Gastroenterol. 22, 501–518. 10.3748/wjg.v22.i2.501 26811603 PMC4716055

[B22] Gao Z. Guo B. Gao R. Zhu Q. Qin H. (2015). Microbiota Disbiosis Is Associated with Colorectal Cancer. Front. Microbiol. 6. 10.3389/fmicb.2015.00020 PMC4313696 25699023

[B23] Goodwin A. C. Shields C. E. D. Wu S. Huso D. L. Wu X. Murray-Stewart T. R. (2011). Polyamine Catabolism Contributes to Enterotoxigenic Bacteroides Fragilis-Induced colon Tumorigenesis. Proc. Natl. Acad. Sci. 108, 15354–15359. 10.1073/pnas.1010203108 21876161 PMC3174648

[B24] Grazioso T. P. Brandt M. Djouder N. (2019). Diet, Microbiota, and Colorectal Cancer. iScience 21, 168–187. 10.1016/j.isci.2019.10.011 31669832 PMC6889474

[B25] Gu W. Deng X. Lee M. Sucu Y. D. Arevalo S. Stryke D. (2021). Rapid Pathogen Detection by Metagenomic Next-Generation Sequencing of Infected Body Fluids. Nat. Med. 27, 115–124. 10.1038/s41591-020-1105-z 33169017 PMC9020267

[B26] Guan R. Wang M. Guan Z. Jin C.-Y. Lin W. Ji X. (2020). Metabolic Engineering for Glycyrrhetinic Acid Production in Saccharomyces cerevisiae. Front. Bioeng. Biotechnol. 8, 1318. 10.3389/fbioe.2020.588255 PMC7710550 33330420

[B27] Haghi F. Goli E. Mirzaei B. Zeighami H. (2019). The Association between Fecal Enterotoxigenic B. Fragilis with Colorectal Cancer. BMC Cancer 19, 879. 10.1186/s12885-019-6115-1 31488085 PMC6727388

[B28] Hillmann B. Al-Ghalith G. A. Shields-Cutler R. R. Zhu Q. Gohl D. M. Beckman K. B. (2018). Evaluating the Information Content of Shallow Shotgun Metagenomics. mSystems 3. 10.1128/mSystems.00069-18 PMC6234283 30443602

[B29] Huerta-Cepas J. Forslund K. Coelho L. P. Szklarczyk D. Jensen L. J. Von Mering C. (2017). Fast Genome-Wide Functional Annotation through Orthology Assignment by eggNOG-Mapper. Mol. Biol. Evol. 34, 2115–2122. 10.1093/molbev/msx148 28460117 PMC5850834

[B30] Illiano P. Brambilla R. Parolini C. (2020). The Mutual Interplay of Gut Microbiota, Diet and Human Disease. Febs j 287, 833–855. 10.1111/febs.15217 31955527

[B31] Jandhyala S. M. Talukdar R. Subramanyam C. Vuyyuru H. Sasikala M. Nageshwar Reddy D. (2015). Role of the normal Gut Microbiota. World J. Gastroenterol. 21, 8787–8803. 10.3748/wjg.v21.i29.8787 26269668 PMC4528021

[B32] Jiang S. Zhang Y. Jin J. Wu Y. Wei Y. Wang X. (2020). Organic Carbon in a Seepage Face of a Subterranean Estuary: Turnover and Microbial Interrelations. Sci. Total Environ. 725, 138220. 10.1016/j.scitotenv.2020.138220 32302826

[B33] Lagier J.-C. Khelaifia S. Alou M. T. Ndongo S. Dione N. Hugon P. (2016). Culture of Previously Uncultured Members of the Human Gut Microbiota by Culturomics. Nat. Microbiol. 1, 16203. 10.1038/nmicrobiol.2016.203 27819657 PMC12094094

[B34] Laudadio I. Fulci V. Palone F. Stronati L. Cucchiara S. Carissimi C. (2018). Quantitative Assessment of Shotgun Metagenomics and 16S rDNA Amplicon Sequencing in the Study of Human Gut Microbiome. OMICS: A J. Integr. Biol. 22, 248–254. 10.1089/omi.2018.0013 29652573

[B35] Li Y. Wang J. Wang M. Gao Y. Jin C.-Y. Shi X. (2021). Microbial Profiling Identifies Potential Key Drivers in Gastric Cancer Patients. Biotechnol. Biotechnological Equipment 35, 496–503. 10.1080/13102818.2021.1896384

[B36] Liang J. Mai W. Tang J. Wei Y. (2019). Highly Effective Treatment of Petrochemical Wastewater by a Super-sized Industrial Scale Plant with Expanded Granular Sludge Bed Bioreactor and Aerobic Activated Sludge. Chem. Eng. J. 360, 15–23. 10.1016/j.cej.2018.11.167

[B37] Liang J. Mai W. Wang J. Li X. Su M. Du J. (2021). Performance and Microbial Communities of a Novel Integrated Industrial-Scale Pulp and Paper Wastewater Treatment Plant. J. Clean. Prod. 278, 123896. 10.1016/j.jclepro.2020.123896

[B38] Lichtenstein P. Holm N. V. Verkasalo P. K. Iliadou A. Kaprio J. Koskenvuo M. (2000). Environmental and Heritable Factors in the Causation of Cancer - Analyses of Cohorts of Twins from Sweden, Denmark, and Finland. N. Engl. J. Med. 343, 78–85. 10.1056/nejm200007133430201 10891514

[B39] Lopez L. R. Bleich R. M. Arthur J. C. (2021). Microbiota Effects on Carcinogenesis: Initiation, Promotion, and Progression. Annu. Rev. Med. 72, 243–261. 10.1146/annurev-med-080719-091604 33052764 PMC10202020

[B40] Mai W. Hu T. Li C. Wu R. Chen J. Shao Y. (2020). Effective Nitrogen Removal of Wastewater from Vitamin B2 Production by a Potential Anammox Process. J. Water Process Eng. 37, 101515. 10.1016/j.jwpe.2020.101515

[B41] Nagpal S. Singh R. Yadav D. Mande S. S. (2020). MetagenoNets: Comprehensive Inference and Meta-Insights for Microbial Correlation Networks. Nucleic Acids Res. 48, W572–W579. 10.1093/nar/gkaa254 32338757 PMC7319469

[B42] Nurk S. Meleshko D. Korobeynikov A. Pevzner P. A. (2017). metaSPAdes: a New Versatile Metagenomic Assembler. Genome Res. 27, 824–834. 10.1101/gr.213959.116 28298430 PMC5411777

[B43] Peng M. Tabashsum Z. Patel P. Bernhardt C. Biswas C. Meng J. (2020). Prevention of Enteric Bacterial Infections and Modulation of Gut Microbiota with Conjugated Linoleic Acids Producing Lactobacillus in Mice. Gut Microbes 11, 433–452. 10.1080/19490976.2019.1638724 31411526 PMC7524329

[B44] Peterson D. Bonham K. S. Rowland S. Pattanayak C. W. Klepac-Ceraj V. (2021). Comparative Analysis of 16S rRNA Gene and Metagenome Sequencing in Pediatric Gut Microbiomes. bioRxiv. 10.1101/2021.02.20.432118 PMC8320171 34335499

[B45] Pleguezuelos-Manzano C. Puschhof J. Puschhof J. Rosendahl Huber A. van Hoeck A. Wood H. M. (2020). Mutational Signature in Colorectal Cancer Caused by Genotoxic Pks+ E. coli. Nature 580, 269–273. 10.1038/s41586-020-2080-8 32106218 PMC8142898

[B46] Qiao S. Wu D. Wang M. Qian S. Zhu Y. Shi J. (2020). Oral Microbial Profile Variation during Canine Ligature-Induced Peri-Implantitis Development. BMC Microbiol. 20, 293. 10.1186/s12866-020-01982-6 32993514 PMC7526148

[B47] Rajamäki K. Taira A. Katainen R. Välimäki N. Kuosmanen A. Plaketti R. M. (2021). Genetic and Epigenetic Characteristics of Inflammatory Bowel Disease Associated Colorectal Cancer. Gastroenterology. 10.1053/j.gastro.2021.04.042 33930428

[B48] Rausch P. Rühlemann M. Hermes B. M. Doms S. Dagan T. Dierking K. (2019). Comparative Analysis of Amplicon and Metagenomic Sequencing Methods Reveals Key Features in the Evolution of Animal Metaorganisms. Microbiome 7 (1), 133. 10.1186/s40168-019-0743-1 31521200 PMC6744666

[B49] Reis S. A. d. da Conceição L. L. Peluzio M. d. C. G. (2019). Intestinal Microbiota and Colorectal Cancer: Changes in the Intestinal Microenvironment and Their Relation to the Disease. J. Med. Microbiol. 68, 1391–1407. 10.1099/jmm.0.001049 31424382

[B50] Rubinstein M. R. Baik J. E. Lagana S. M. Han R. P. Raab W. J. Sahoo D. (2019). Fusobacterium Nucleatum Promotes Colorectal Cancer by Inducing Wnt/β-Catenin Modulator Annexin A1. EMBO Rep. 20. 10.15252/embr.201847638 PMC6446206 30833345

[B51] Russo E. Bacci G. Chiellini C. Fagorzi C. Niccolai E. Taddei A. (2017). Preliminary Comparison of Oral and Intestinal Human Microbiota in Patients with Colorectal Cancer: A Pilot Study. Front. Microbiol. 8, 2699. 10.3389/fmicb.2017.02699 29375539 PMC5770402

[B52] Saito K. Koido S. Odamaki T. Kajihara M. Kato K. Horiuchi S. (2019). Metagenomic Analyses of the Gut Microbiota Associated with Colorectal Adenoma. PLoS One 14, e0212406. 10.1371/journal.pone.0212406 30794590 PMC6386391

[B53] Santiago-Rodriguez T. M. Garoutte A. Adams E. Nasser W. Ross M. C. La Reau A. (2020). Metagenomic Information Recovery from Human Stool Samples Is Influenced by Sequencing Depth and Profiling Method. Genes (Basel) 11. 10.3390/genes11111380 PMC7700633 33233349

[B54] Schmitt M. Greten F. R. (2021). The Inflammatory Pathogenesis of Colorectal Cancer. Nat. Rev. Immunol. 10.1038/s41577-021-00534-x 33911231

[B55] Sears C. L. Geis A. L. Housseau F. (2014). Bacteroides Fragilis Subverts Mucosal Biology: from Symbiont to colon Carcinogenesis. J. Clin. Invest. 124, 4166–4172. 10.1172/jci72334 25105360 PMC4191034

[B56] Shang F.-M. Liu H.-L. (2018). Fusobacterium Nucleatumand Colorectal Cancer: A Review. World J. Gastrointest. Oncol. 10, 71–81. 10.4251/wjgo.v10.i3.71 29564037 PMC5852398

[B57] Shi X.-J. Wei Y. Ji B. (2020). Systems Biology of Gastric Cancer: Perspectives on the Omics-Based Diagnosis and Treatment. Front. Mol. Biosci. 7, 203. 10.3389/fmolb.2020.00203 33005629 PMC7479200

[B58] Song M. Chan A. T. (2019). Environmental Factors, Gut Microbiota, and Colorectal Cancer Prevention. Clin. Gastroenterol. Hepatol. 17, 275–289. 10.1016/j.cgh.2018.07.012 30031175 PMC6314893

[B59] Song M. Chan A. T. Sun J. (2020). Influence of the Gut Microbiome, Diet, and Environment on Risk of Colorectal Cancer. Gastroenterology 158, 322–340. 10.1053/j.gastro.2019.06.048 31586566 PMC6957737

[B60] Sung H. Ferlay J. Siegel R. L. Laversanne M. Soerjomataram I. Jemal A. (2021). Global Cancer Statistics 2020: GLOBOCAN Estimates of Incidence and Mortality Worldwide for 36 Cancers in 185 Countries. CA Cancer J. Clin. 71 (3), 209–249. 10.3322/caac.21660 33538338

[B61] Tett A. Huang K. D. Asnicar F. Fehlner-Peach H. Pasolli E. Karcher N. (2019). The Prevotella Copri Complex Comprises Four Distinct Clades Underrepresented in Westernized Populations. Cell Host & Microbe 26, 666–679. 10.1016/j.chom.2019.08.018 31607556 PMC6854460

[B62] Tilg H. Adolph T. E. Gerner R. R. Moschen A. R. (2018). The Intestinal Microbiota in Colorectal Cancer. Cancer Cell 33, 954–964. 10.1016/j.ccell.2018.03.004 29657127

[B63] Tjalsma H. Boleij A. Marchesi J. R. Dutilh B. E. (2012). A Bacterial Driver-Passenger Model for Colorectal Cancer: beyond the Usual Suspects. Nat. Rev. Microbiol. 10, 575–582. 10.1038/nrmicro2819 22728587

[B64] Verhaar B. J. H. Collard D. Prodan A. Levels J. H. M. Zwinderman A. H. Bäckhed F. (2020). Associations between Gut Microbiota, Faecal Short-Chain Fatty Acids, and Blood Pressure across Ethnic Groups: the HELIUS Study. Eur. Heart J. 41, 4259–4267. 10.1093/eurheartj/ehaa704 32869053 PMC7724641

[B65] Wang B. Yao M. Lv L. Ling Z. Li L. (2017). The Human Microbiota in Health and Disease. Engineering 3, 71–82. 10.1016/j.eng.2017.01.008

[B66] Wang M. Wei Y. Ji B. Nielsen J. (2020). Advances in Metabolic Engineering of Saccharomyces cerevisiae for Cocoa Butter Equivalent Production. Front. Bioeng. Biotechnol. 8. 10.3389/fbioe.2020.594081 PMC7594527 33178680

[B67] Wang W.-L. Xu S.-Y. Ren Z.-G. Tao L. Jiang J.-W. Zheng S.-S. (2015). Application of Metagenomics in the Human Gut Microbiome. World J. Gastroenterol. 21, 803–814. 10.3748/wjg.v21.i3.803 25624713 PMC4299332

[B68] Wassenaar T. M. (2018). E. coli and Colorectal Cancer: a Complex Relationship that Deserves a Critical Mindset. Crit. Rev. Microbiol. 44, 619–632. 10.1080/1040841x.2018.1481013 29909724

[B69] Wei Y. Ji B. Siewers V. Xu D. Halkier B. A. Nielsen J. (2019). Identification of Genes Involved in Shea Butter Biosynthesis from Vitellaria Paradoxa Fruits through Transcriptomics and Functional Heterologous Expression. Appl. Microbiol. Biotechnol. 103, 3727–3736. 10.1007/s00253-019-09720-3 30915502 PMC6469615

[B70] Wei Y. Ren T. Zhang L. (2020a). Dix-seq: An Integrated Pipeline for Fast Amplicon Data Analysis. bioRxiv. 10.1101/2020.05.11.089748,

[B71] Wei Y. Wu Y. Zhang L. Zhou Z. Zhou H. Yan X. (2020b). Genome Recovery and Metatranscriptomic Confirmation of Functional Acetate-Oxidizing Bacteria from Enriched Anaerobic Biogas Digesters. Environ. Pollut., 265, 114843. 10.1016/j.envpol.2020.114843 32480003

[B72] Wirbel J. Pyl P. T. Kartal E. Zych K. Kashani A. Milanese A. (2019). Meta-analysis of Fecal Metagenomes Reveals Global Microbial Signatures that Are Specific for Colorectal Cancer. Nat. Med. 25, 679–689. 10.1038/s41591-019-0406-6 30936547 PMC7984229

[B73] Wong S. H. Yu J. (2019). Gut Microbiota in Colorectal Cancer: Mechanisms of Action and Clinical Applications. Nat. Rev. Gastroenterol. Hepatol. 16, 690–704. 10.1038/s41575-019-0209-8 31554963

[B74] Xing C. Wang M. Ajibade A. A. Tan P. Fu C. Chen L. (2021). Microbiota Regulate Innate Immune Signaling and Protective Immunity against Cancer. Cell Host Microbe 29 (6), 959–974. 10.1016/j.chom.2021.03.016 33894128 PMC8192480

[B75] Yachida S. Mizutani S. Shiroma H. Shiba S. Nakajima T. Sakamoto T. (2019). Metagenomic and Metabolomic Analyses Reveal Distinct Stage-specific Phenotypes of the Gut Microbiota in Colorectal Cancer. Nat. Med. 25, 968–976. 10.1038/s41591-019-0458-7 31171880

[B76] Yamashita Y. Takeshita T. (2017). The Oral Microbiome and Human Health. J. Oral Sci. 59, 201–206. 10.2334/josnusd.16-0856 28637979

[B77] Zhu Q. Huang S. Gonzalez A. Mcgrath I. Mcdonald D. Haiminen N. (2021). OGUs Enable Effective, Phylogeny-Aware Analysis of Even Shallow Metagenome Community Structures. bioRxiv. 10.1101/2021.04.04.438427

