# Supplementary material for: Exploring Gut Microbiota in Patients with Colorectal Disease Based on 16S rRNA Gene Amplicon and Shallow Metagenomic Sequencing
Source: Front Mol Biosci. 2021 Jul 9;8:703638. doi: 10.3389/fmolb.2021.703638 (PMC8299945; doi:10.3389/fmolb.2021.703638)
Supplement: Supplementary file 1 [file DataSheet1.docx]

**Exploring gut microbiota in patients with colorectal disease based on 16S rRNA gene amplicon and shallow metagenomic sequencing**

**Running title: Colorectal disease patients’ gut microbiota**

Yuanfeng Liu^1^, Xiang Li^2^, Yudie Yang^4,6^, Ye Liu^5^, Shijun Wang^5^, Boyang Ji^3^, Yongjun Wei^4,6*^

^1^ Department of Vascular and Endovascular Surgery, First Affiliated Hospital of Zhengzhou University, Zhengzhou 450052, Henan, PR China

^2^ Science China Press, Beijing 100171, China

^3^ Department of Biology and Biological Engineering, Chalmers University of Technology, SE412 96 Gothenburg, Sweden

^4^ Key Laboratory of Advanced Drug Preparation Technologies, School of Pharmaceutical Sciences, Ministry of Education, Zhengzhou University, Zhengzhou 450052, PR China

^5^ Oncology Department, Colorectal and anal surgery department, First Affiliated Hospital of Zhengzhou University, Zhengzhou 450052, Henan, PR China

^6^ Laboratory of Synthetic Biology, Zhengzhou University, Zhengzhou 450052, PR China

Corresponding author

Yongjun Wei

Email: [yongjunwei@zzu.edu.cn](mailto:yongjunwei@zzu.edu.cn)


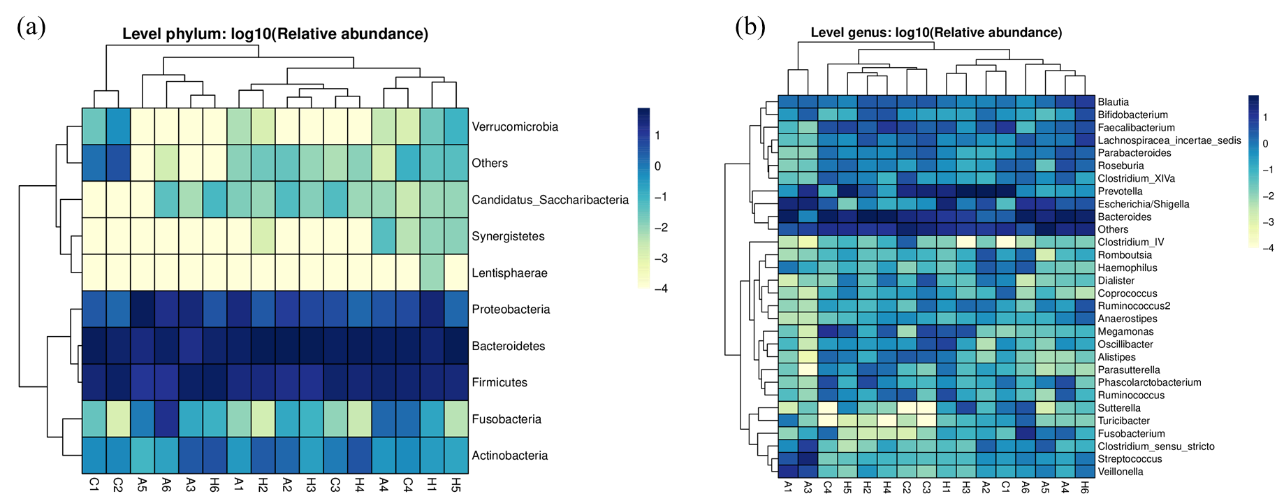


Figure S1 Heatmap microbial distribution of the samples based on 16S rRNA gene amplicon data at (a) phylum- and (b) genus- level.


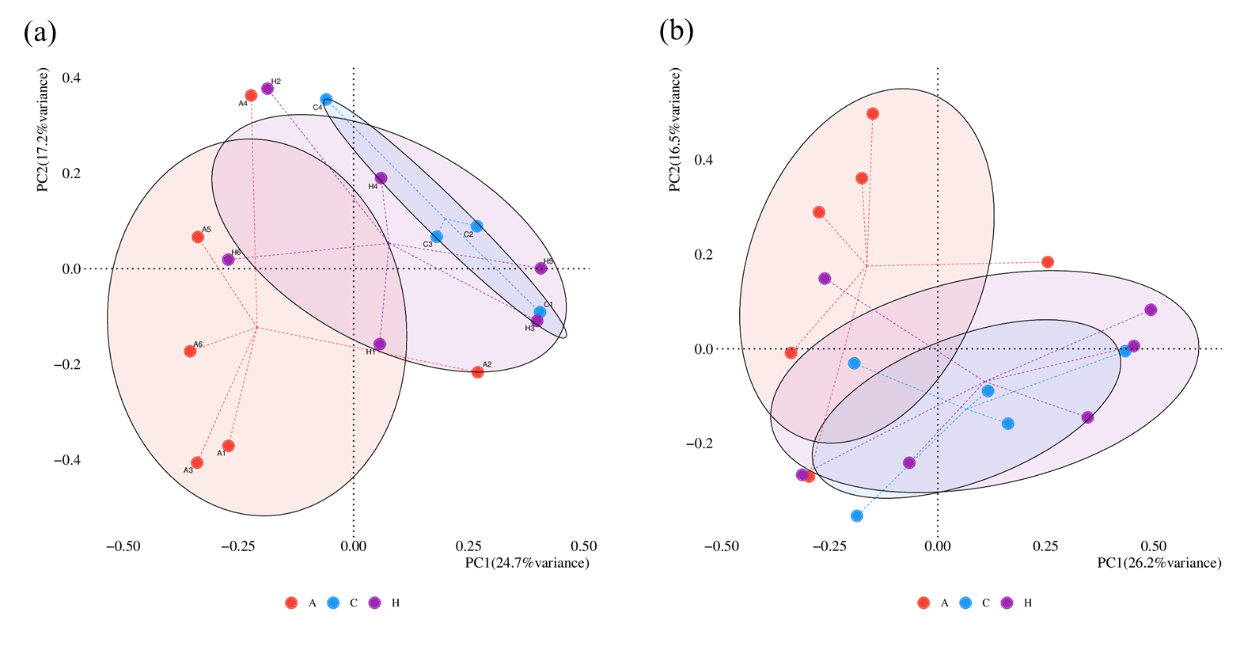


Figure S2 PCoA analyses of the three groups based on bray curtis distances with (a) 16S rRNA gene amplicon data and (b) SMS data.

Table S1 Samples used in this study.

| Samples | Gender | Age | Type of symptom | Sequence numbers | Groups |
| --- | --- | --- | --- | --- | --- |
| A1 | Male | 72 | Adenoma | 177053 | A |
| A2 | Male | 48 | Adenoma | 46901 | A |
| A3 | Male | 72 | Adenoma | 236821 | A |
| A4 | Female | 49 | Adenoma | 139389 | A |
| A5 | Female | 63 | Adenoma | 73641 | A |
| A6 | Male | 51 | Adenoma | 118857 | A |
| C1 | Female | 65 | Colorectal cancer | 18667 | C |
| C2 | Female | 50 | Colorectal cancer | 78835 | C |
| C3 | Male | 71 | Colorectal cancer | 18965 | C |
| C4 | Male | 57 | Colorectal cancer | 84162 | C |
| H1 | Male | 24 | Hemorrhoids | 137935 | H |
| H2 | Female | 64 | Hemorrhoids | 167597 | H |
| H3 | Male | 32 | Hemorrhoids | 23085 | H |
| H4 | Male | 24 | Hemorrhoids | 40626 | H |
| H5 | Female | 29 | Hemorrhoids | 73972 | H |
| H6 | Female | 49 | Hemorrhoids | 52460 | H |

Table S2 SMS data of the samples.

|  | Sequence read number | Sequence length (bp) | Q20(%) | Q30(%) |
| --- | --- | --- | --- | --- |
| A1 | 8568105 | 2.57 G | 97.4125 | 92.5507 |
| A2 | 4141100 | 1.24 G | 97.2753 | 92.3912 |
| A3 | 1497066 | 0.45 G | 96.8708 | 91.7283 |
| A4 | 3255829 | 0.98 G | 97.2714 | 92.3913 |
| A5 | 2839997 | 0.85 G | 97.0464 | 91.9758 |
| A6 | 12566683 | 3.77 G | 96.6407 | 91.0695 |
| C1 | 1919555 | 5.76 G | 96.7296 | 91.3995 |
| C2 | 8389629 | 2.52 G | 97.3299 | 92.5073 |
| C3 | 4168536 | 1.25 G | 97.5553 | 92.9779 |
| C4 | 10210826 | 3.06 G | 97.3902 | 92.5038 |
| H1 | 1772999 | 0.53 G | 97.2125 | 92.2778 |
| H2 | 1822930 | 0.55 G | 96.9988 | 91.818 |
| H3 | 8220340 | 2.47G | 97.1792 | 92.179 |
| H4 | 11615232 | 3.48G | 97.6395 | 93.1473 |
| H5 | 1863208 | 0.56 G | 97.2148 | 92.2457 |
| H6 | 2515138 | 0.75 G | 96.6998 | 91.2103 |

Table S3 Alpha diversity of the samples based on SMS data.

|  | A1 | A2 | A3 | A4 | A5 | A6 | C1 | C2 | C3 | C4 | H1 | H2 | H3 | H4 | H5 | H6 | Group A | Group C | Group H |
| --- | --- | --- | --- | --- | --- | --- | --- | --- | --- | --- | --- | --- | --- | --- | --- | --- | --- | --- | --- |
| Richness | 576 | 854 | 70 | 357 | 223 | 907 | 463 | 1493 | 598 | 961 | 355 | 253 | 761 | 1077 | 430 | 307 | 497.83 | 878.75 | 530.5 |
| Chao1 | 576 | 854 | 70 | 357 | 223 | 907 | 463 | 1493 | 598 | 961 | 355 | 253 | 761 | 1077 | 430 | 307 | 497.83 | 878.75 | 530.5 |
| Shannon_2 | 2.84 | 4.38 | 3.27 | 4.49 | 3.81 | 4.03 | 4.12 | 6.36 | 5.38 | 5.3 | 3.04 | 3.94 | 2.25 | 4.95 | 4.84 | 3.94 | 3.80 | 5.29 | 3.83 |
| Simpson | 0.32 | 0.192 | 0.21 | 0.12 | 0.17 | 0.16 | 0.25 | 0.038 | 0.076 | 0.078 | 0.40 | 0.14 | 0.55 | 0.11 | 0.14 | 0.19 | 0.20 | 0.11 | 0.25 |
| Dominance | 0.68 | 0.808 | 0.79 | 0.88 | 0.83 | 0.84 | 0.75 | 0.96 | 0.92 | 0.92 | 0.60 | 0.86 | 0.449 | 0.89 | 0.86 | 0.81 | 0.80 | 0.89 | 0.75 |
| Equitability | 0.31 | 0.45 | 0.53 | 0.53 | 0.49 | 0.41 | 0.47 | 0.60 | 0.58 | 0.54 | 0.36 | 0.49 | 0.24 | 0.49 | 0.55 | 0.48 | 0.45 | 0.55 | 0.43 |

Table S4 Microbial distribution at phylum-level based on SMS data.

|  | A1 | A2 | A3 | A4 | A5 | A6 | C1 | C2 | C3 | C4 | H1 | H2 | H3 | H4 | H5 | H6 | Group A | Group C | Group H |
| --- | --- | --- | --- | --- | --- | --- | --- | --- | --- | --- | --- | --- | --- | --- | --- | --- | --- | --- | --- |
| *Bacteroidota* | 57.02% | 68.66% | 7.50% | 69.58% | 34.35% | 61.44% | 71.02% | 37.02% | 66.54% | 72.04% | 83.07% | 81.21% | 92.51% | 72.78% | 70.67% | 54.50% | 49.76% | 61.66% | 75.79% |
| *Firmicutes*_A | 1.46% | 10.08% | 7.91% | 21.47% | 7.72% | 11.80% | 18.51% | 55.32% | 26.55% | 18.15% | 6.52% | 11.56% | 2.87% | 21.00% | 23.51% | 35.59% | 10.07% | 29.63% | 16.84% |
| *Proteobacteria* | 26.37% | 15.51% | 45.30% | 2.74% | 54.94% | 16.81% | 2.05% | 1.21% | 1.21% | 3.39% | 1.58% | 1.35% | 1.32% | 1.55% | 1.84% | 2.82% | 26.94% | 1.97% | 1.74% |
| *Firmicutes* | 3.28% | 0.98% | 27.97% | 0.32% | 0.49% | 1.46% | 3.47% | 1.46% | 0.36% | 0.39% | 0.31% | 0.27% | 0.24% | 0.27% | 0.33% | 0.84% | 5.75% | 1.42% | 0.38% |
| *Firmicutes*_C | 10.32% | 1.81% | 2.39% | 1.22% | 0.49% | 0.62% | 2.73% | 0.51% | 2.43% | 2.51% | 6.60% | 2.03% | 1.77% | 1.29% | 1.68% | 1.75% | 2.81% | 2.05% | 2.52% |
| Unclassified | 0.93% | 0.86% | 1.68% | 2.87% | 1.02% | 0.86% | 0.95% | 1.86% | 1.45% | 1.84% | 0.63% | 1.96% | 0.88% | 0.97% | 0.87% | 1.12% | 1.37% | 1.53% | 1.07% |
| *Actinobacteriota* | 0.44% | 1.42% | 6.95% | 0.22% | 0.08% | 0.43% | 0.63% | 0.93% | 0.54% | 0.13% | 0.51% | 1.05% | 0.13% | 1.55% | 0.28% | 3.20% | 1.59% | 0.56% | 1.12% |
| *Fusobacteriota* | 0.02% | 0.12% | 0.10% | 0.44% | 0.75% | 5.63% | 0.03% | 0.02% | 0.02% | 0.75% | 0.04% | 0.01% | 0.02% | 0.01% | 0.02% | 0.01% | 1.18% | 0.20% | 0.02% |
| *Desulfobacterota*_A | 0.00% | 0.01% | 0.00% | 0.89% | 0.10% | 0.70% | 0.03% | 0.13% | 0.50% | 0.47% | 0.34% | 0.25% | 0.01% | 0.37% | 0.33% | 0.01% | 0.28% | 0.28% | 0.22% |
| Other phyla | 0.16% | 0.55% | 0.20% | 0.26% | 0.06% | 0.24% | 0.58% | 1.53% | 0.41% | 0.33% | 0.40% | 0.32% | 0.26% | 0.23% | 0.48% | 0.16% | 0.24% | 0.71% | 0.31% |

Table S5 Microbial distribution at genus-level based on SMS data.

|  | A1 | A2 | A3 | A4 | A5 | A6 | C1 | C2 | C3 | C4 | H1 | H2 | H3 | H4 | H5 | H6 | Group A | Group C | Group H |
| --- | --- | --- | --- | --- | --- | --- | --- | --- | --- | --- | --- | --- | --- | --- | --- | --- | --- | --- | --- |
| Prevotella | 0.22% | 61.29% | 6.78% | 0.15% | 0.06% | 0.68% | 62.86% | 14.25% | 19.06% | 0.23% | 70.98% | 0.15% | 84.29% | 0.28% | 37.86% | 0.04% | 11.53% | 24.10% | 32.27% |
| Bacteroides_B | 0.83% | 0.90% | 0.03% | 23.52% | 15.86% | 28.75% | 0.87% | 1.95% | 18.75% | 16.84% | 2.09% | 26.34% | 0.57% | 3.87% | 2.06% | 36.37% | 11.65% | 9.60% | 11.88% |
| Bacteroides | 1.68% | 1.11% | 0.11% | 31.58% | 12.86% | 26.95% | 2.20% | 4.77% | 9.10% | 17.84% | 2.30% | 40.92% | 1.10% | 8.86% | 3.33% | 9.08% | 12.38% | 8.48% | 10.93% |
| Bacteroides_A | 49.46% | 2.17% | 0.08% | 0.92% | 0.06% | 0.05% | 0.91% | 7.61% | 6.69% | 23.48% | 3.36% | 0.36% | 3.24% | 46.22% | 18.96% | 0.09% | 8.79% | 9.67% | 12.04% |
| Unclassified | 7.22% | 4.07% | 6.02% | 12.44% | 7.20% | 5.85% | 3.84% | 7.38% | 8.57% | 9.40% | 3.52% | 14.05% | 3.51% | 7.52% | 5.01% | 9.12% | 7.13% | 7.30% | 7.12% |
| Escherichia | 23.03% | 6.07% | 40.40% | 1.86% | 7.18% | 10.66% | 0.03% | 0.03% | 0.16% | 1.46% | 0.13% | 0.23% | 0.07% | 0.01% | 0.08% | 2.41% | 14.87% | 0.42% | 0.49% |
| Faecalibacterium | 0.00% | 4.73% | 0.02% | 1.36% | 1.22% | 0.18% | 4.68% | 7.04% | 5.58% | 2.88% | 0.98% | 1.12% | 0.01% | 8.07% | 5.62% | 4.12% | 1.25% | 5.05% | 3.32% |
| Klebsiella | 0.01% | 0.11% | 0.26% | 0.01% | 37.06% | 0.08% | 0.01% | 0.00% | 0.01% | 0.01% | 0.00% | 0.01% | 0.01% | 0.01% | 0.01% | 0.00% | 6.25% | 0.01% | 0.01% |
| Streptococcus | 1.85% | 0.41% | 22.66% | 0.03% | 0.21% | 0.22% | 0.15% | 0.07% | 0.04% | 0.03% | 0.05% | 0.07% | 0.03% | 0.07% | 0.03% | 0.45% | 4.23% | 0.07% | 0.12% |
| Parabacteroides | 0.10% | 0.08% | 0.01% | 4.00% | 1.61% | 1.35% | 0.34% | 1.43% | 2.40% | 2.27% | 0.76% | 1.18% | 0.11% | 1.44% | 0.97% | 3.15% | 1.19% | 1.61% | 1.27% |
| Lachnospira | 0.00% | 0.32% | 0.01% | 2.54% | 0.01% | 0.05% | 0.85% | 0.44% | 2.19% | 0.38% | 0.28% | 1.52% | 0.37% | 0.87% | 4.65% | 3.59% | 0.49% | 0.97% | 1.88% |
| Blautia_A | 0.01% | 0.21% | 1.57% | 2.05% | 0.61% | 0.21% | 0.58% | 0.92% | 1.21% | 0.51% | 0.39% | 1.37% | 0.13% | 1.16% | 0.51% | 5.38% | 0.78% | 0.81% | 1.49% |
| Clostridium_M | 0.00% | 0.23% | 0.01% | 2.40% | 0.23% | 1.05% | 0.09% | 1.07% | 0.54% | 1.70% | 0.78% | 2.25% | 0.65% | 0.19% | 0.22% | 5.11% | 0.65% | 0.85% | 1.53% |
| Alistipes | 0.03% | 0.02% | 0.01% | 0.02% | 0.01% | 0.07% | 0.58% | 2.45% | 2.17% | 2.85% | 0.29% | 0.37% | 0.01% | 3.30% | 2.20% | 0.06% | 0.02% | 2.01% | 1.04% |
| Veillonella | 10.30% | 0.28% | 2.32% | 0.02% | 0.26% | 0.47% | 0.18% | 0.00% | 0.00% | 0.01% | 0.00% | 0.20% | 0.02% | 0.02% | 0.01% | 0.04% | 2.27% | 0.05% | 0.05% |
| Faecalicatena | 0.01% | 0.88% | 0.10% | 0.45% | 1.22% | 2.58% | 0.16% | 0.17% | 0.58% | 0.52% | 0.18% | 0.26% | 0.56% | 0.19% | 0.16% | 4.69% | 0.87% | 0.36% | 1.00% |
| Megamonas | 0.00% | 0.00% | 0.00% | 0.00% | 0.00% | 0.00% | 0.00% | 0.00% | 1.32% | 1.54% | 6.18% | 0.00% | 1.69% | 0.40% | 0.80% | 0.00% | 0.00% | 0.71% | 1.51% |
| Bifidobacterium | 0.27% | 0.25% | 5.04% | 0.07% | 0.00% | 0.02% | 0.20% | 0.23% | 0.03% | 0.01% | 0.09% | 0.84% | 0.02% | 1.13% | 0.01% | 3.06% | 0.94% | 0.12% | 0.86% |
| Agathobacter | 0.00% | 0.04% | 0.01% | 1.87% | 0.01% | 0.08% | 1.35% | 0.95% | 0.20% | 0.81% | 0.29% | 0.03% | 0.10% | 1.34% | 0.33% | 1.85% | 0.33% | 0.83% | 0.66% |
| GCA-900066995 | 0.00% | 0.00% | 0.00% | 0.02% | 0.00% | 0.00% | 0.01% | 7.54% | 0.01% | 0.01% | 0.00% | 0.00% | 0.00% | 0.01% | 0.01% | 0.00% | 0.00% | 1.89% | 0.00% |
| Roseburia | 0.00% | 0.04% | 0.01% | 2.32% | 0.01% | 1.08% | 0.68% | 0.18% | 0.58% | 0.85% | 0.13% | 0.33% | 0.02% | 0.18% | 0.97% | 0.12% | 0.58% | 0.57% | 0.29% |
| Fusobacterium_A | 0.00% | 0.00% | 0.00% | 0.43% | 0.74% | 5.41% | 0.00% | 0.00% | 0.00% | 0.63% | 0.02% | 0.00% | 0.00% | 0.00% | 0.00% | 0.00% | 1.10% | 0.16% | 0.00% |
| Citrobacter | 0.00% | 0.00% | 0.01% | 0.00% | 5.15% | 0.01% | 0.49% | 0.00% | 0.02% | 0.81% | 0.10% | 0.00% | 0.01% | 0.00% | 0.00% | 0.00% | 0.86% | 0.33% | 0.02% |

Table S6 Microbial distribution at species-level based on SMS data.

|  | A1 | A2 | A3 | A4 | A5 | A6 | C1 | C2 | C3 | C4 | H1 | H2 | H3 | H4 | H5 | H6 | Group A | Group C | Group H |
| --- | --- | --- | --- | --- | --- | --- | --- | --- | --- | --- | --- | --- | --- | --- | --- | --- | --- | --- | --- |
| Unclassified | 27.87% | 11.71% | 39.69% | 21.02% | 23.00% | 18.39% | 6.46% | 10.52% | 13.43% | 16.37% | 5.64% | 22.80% | 5.72% | 11.91% | 7.70% | 16.89% | 23.61% | 11.70% | 11.78% |
| Prevotella copri | 0.04% | 15.84% | 0.90% | 0.01% | 0.00% | 0.23% | 45.66% | 11.88% | 16.74% | 0.01% | 59.33% | 0.01% | 70.55% | 0.04% | 33.68% | 0.00% | 2.84% | 18.57% | 27.27% |
| Bacteroides_B dorei | 0.22% | 0.07% | 0.01% | 0.19% | 3.91% | 25.10% | 0.12% | 0.05% | 13.90% | 0.44% | 0.77% | 3.83% | 0.03% | 0.14% | 0.11% | 32.77% | 4.92% | 3.62% | 6.28% |
| Bacteroides_A coprocola | 48.34% | 0.10% | 0.03% | 0.02% | 0.01% | 0.01% | 0.01% | 3.08% | 0.35% | 3.55% | 0.03% | 0.03% | 1.21% | 16.09% | 6.25% | 0.00% | 8.08% | 1.75% | 3.93% |
| Bacteroides_B vulgatus | 0.26% | 0.69% | 0.01% | 21.01% | 10.22% | 0.97% | 0.62% | 0.84% | 0.87% | 14.33% | 0.14% | 18.30% | 0.41% | 3.10% | 1.63% | 0.27% | 5.53% | 4.16% | 3.98% |
| Bacteroides_A plebeius_A | 0.03% | 1.48% | 0.04% | 0.03% | 0.00% | 0.01% | 0.11% | 2.98% | 1.87% | 15.06% | 2.52% | 0.02% | 1.41% | 23.03% | 6.44% | 0.01% | 0.26% | 5.00% | 5.57% |
| Prevotella copri_A | 0.00% | 36.02% | 0.36% | 0.01% | 0.00% | 0.15% | 1.68% | 0.58% | 0.71% | 0.02% | 2.02% | 0.00% | 6.42% | 0.03% | 1.12% | 0.00% | 6.09% | 0.75% | 1.60% |
| Bacteroides uniformis | 0.06% | 0.12% | 0.01% | 10.89% | 0.91% | 0.41% | 0.49% | 0.79% | 2.25% | 3.41% | 0.63% | 7.82% | 0.09% | 3.27% | 0.52% | 0.05% | 2.07% | 1.74% | 2.06% |
| Klebsiella quasipneumoniae | 0.00% | 0.01% | 0.00% | 0.00% | 29.17% | 0.00% | 0.00% | 0.00% | 0.00% | 0.00% | 0.00% | 0.00% | 0.00% | 0.00% | 0.00% | 0.00% | 4.86% | 0.00% | 0.00% |
| Bacteroides fragilis | 0.06% | 0.12% | 0.00% | 0.83% | 0.08% | 22.64% | 0.03% | 0.06% | 0.18% | 0.40% | 0.09% | 0.26% | 0.05% | 0.16% | 0.08% | 1.94% | 3.96% | 0.17% | 0.43% |
| Bacteroides stercoris | 0.24% | 0.10% | 0.01% | 0.04% | 2.82% | 0.04% | 0.17% | 0.35% | 0.04% | 0.11% | 0.46% | 15.85% | 0.02% | 0.83% | 0.31% | 0.09% | 0.54% | 0.17% | 2.93% |
| Bacteroides ovatus | 0.09% | 0.12% | 0.01% | 0.69% | 3.31% | 0.10% | 0.25% | 0.23% | 0.23% | 1.17% | 0.16% | 8.16% | 0.15% | 0.20% | 0.13% | 4.39% | 0.72% | 0.47% | 2.20% |
| Streptococcus pasteurianus | 0.00% | 0.00% | 18.48% | 0.00% | 0.00% | 0.01% | 0.00% | 0.00% | 0.00% | 0.00% | 0.00% | 0.00% | 0.00% | 0.00% | 0.00% | 0.00% | 3.08% | 0.00% | 0.00% |
| Escherichia coli_D | 3.90% | 1.86% | 10.05% | 0.37% | 0.03% | 0.75% | 0.00% | 0.00% | 0.04% | 0.05% | 0.02% | 0.07% | 0.01% | 0.00% | 0.00% | 0.60% | 2.83% | 0.02% | 0.11% |
| Parabacteroides distasonis | 0.03% | 0.05% | 0.01% | 3.83% | 1.34% | 1.32% | 0.21% | 0.90% | 1.17% | 1.16% | 0.32% | 0.94% | 0.08% | 0.68% | 0.29% | 2.93% | 1.10% | 0.86% | 0.88% |
| Megamonas funiformis | 0.00% | 0.00% | 0.00% | 0.00% | 0.00% | 0.00% | 0.00% | 0.00% | 1.31% | 1.53% | 6.15% | 0.00% | 1.67% | 0.40% | 0.80% | 0.00% | 0.00% | 0.71% | 1.50% |
| Bacteroides_A plebeius | 0.05% | 0.30% | 0.01% | 0.01% | 0.02% | 0.00% | 0.02% | 0.63% | 0.43% | 2.82% | 0.51% | 0.07% | 0.29% | 4.96% | 1.45% | 0.00% | 0.07% | 0.98% | 1.21% |
| Prevotella sp002265625 | 0.00% | 0.50% | 0.06% | 0.00% | 0.00% | 0.00% | 10.26% | 0.09% | 0.02% | 0.00% | 0.18% | 0.01% | 0.22% | 0.01% | 0.11% | 0.00% | 0.10% | 2.59% | 0.09% |
| Clostridium_M sp000431375 | 0.00% | 0.19% | 0.00% | 0.98% | 0.00% | 0.05% | 0.05% | 0.81% | 0.37% | 0.90% | 0.63% | 1.78% | 0.52% | 0.10% | 0.14% | 4.84% | 0.20% | 0.53% | 1.33% |
| Faecalibacterium prausnitzii_G | 0.00% | 0.31% | 0.00% | 1.02% | 0.33% | 0.03% | 0.69% | 1.02% | 0.77% | 0.43% | 0.07% | 0.66% | 0.00% | 1.16% | 0.69% | 3.39% | 0.28% | 0.73% | 0.99% |
| Bacteroides faecis | 0.01% | 0.18% | 0.02% | 6.40% | 0.02% | 0.01% | 0.27% | 0.05% | 0.91% | 0.04% | 0.04% | 1.38% | 0.07% | 0.40% | 0.31% | 0.03% | 1.11% | 0.32% | 0.37% |
| Faecalibacterium prausnitzii_D | 0.00% | 1.03% | 0.01% | 0.04% | 0.03% | 0.02% | 2.15% | 0.45% | 0.26% | 0.09% | 0.42% | 0.08% | 0.00% | 2.84% | 2.52% | 0.09% | 0.19% | 0.74% | 0.99% |
| Blautia_A wexlerae | 0.01% | 0.09% | 1.08% | 1.05% | 0.50% | 0.11% | 0.11% | 0.14% | 0.51% | 0.26% | 0.24% | 0.70% | 0.05% | 0.70% | 0.13% | 3.99% | 0.47% | 0.26% | 0.97% |
| Bacteroides thetaiotaomicron | 0.02% | 0.08% | 0.01% | 0.29% | 2.72% | 2.01% | 0.20% | 0.30% | 0.08% | 1.52% | 0.12% | 0.62% | 0.12% | 0.13% | 0.11% | 0.64% | 0.86% | 0.52% | 0.29% |
| Faecalibacterium prausnitzii_K | 0.00% | 0.32% | 0.00% | 0.05% | 0.04% | 0.04% | 0.36% | 3.54% | 2.17% | 0.61% | 0.12% | 0.05% | 0.00% | 0.47% | 0.64% | 0.09% | 0.08% | 1.67% | 0.23% |
| Lachnospira rogosae | 0.00% | 0.26% | 0.00% | 2.34% | 0.00% | 0.00% | 0.32% | 0.02% | 1.07% | 0.01% | 0.23% | 0.01% | 0.35% | 0.32% | 3.23% | 0.04% | 0.44% | 0.35% | 0.70% |
| Faecalibacterium prausnitzii_C | 0.00% | 2.43% | 0.00% | 0.04% | 0.69% | 0.04% | 0.28% | 0.27% | 0.54% | 1.01% | 0.20% | 0.06% | 0.00% | 1.80% | 0.65% | 0.12% | 0.53% | 0.53% | 0.47% |
| Total | 81.24% | 73.99% | 70.81% | 71.17% | 79.17% | 72.45% | 70.52% | 39.57% | 60.22% | 65.28% | 81.04% | 83.51% | 89.46% | 72.77% | 69.04% | 73.17% | 74.81% | 58.90% | 78.16% |

Table S7 The global network property of the dominant OTUs in these three groups.

| Global Property | A | C | H |
| --- | --- | --- | --- |
| Nodes | 54 | 54 | 54 |
| Edges | 247 | 401 | 241 |
| Diameter | 8 | 3 | 7 |
| Density | 0.16633 | 0.270034 | 0.16229 |
| Avg. Degree | 8.98182 | 14.5818 | 8.76364 |

Table S8 Assembly results of the SMS data.

|  | contigs (>= 0 bp) | contigs (>= 1 Kb) | contigs (>= 5 Kb) | contigs (>= 10 Kb) | contigs (>= 25 Kb) | contigs (>= 50 Kb) | Total length (>= 0 bp) | Total length (>= 1 Kb) | Total length (>= 5 Kb) | Total length (>= 10 Kb) | Total length (>= 25 Kb) | Total length (>= 50 Kb) | contigs | Largest contig | Total length  (bp) | GC (%) | N50 |
| --- | --- | --- | --- | --- | --- | --- | --- | --- | --- | --- | --- | --- | --- | --- | --- | --- | --- |
| A1 | 51409 | 5572 | 1053 | 557 | 229 | 75 | 43316409 | 28155764 | 19164790 | 15766693 | 10863721 | 5409813 | 11207 | 151305 | 32004207 | 41.95 | 9593 |
| A2 | 134728 | 7985 | 1009 | 466 | 156 | 63 | 72003866 | 29009006 | 16838966 | 13046344 | 8315085 | 5156747 | 25046 | 199928 | 40558803 | 49.56 | 2586 |
| A3 | 20034 | 1643 | 446 | 159 | 25 | 3 | 13982699 | 7526587 | 4804295 | 2822605 | 879622 | 161867 | 4424 | 57290 | 9376384 | 45.69 | 5203 |
| A4 | 124748 | 13905 | 1544 | 768 | 335 | 140 | 95301662 | 53424130 | 30944343 | 25614046 | 18852511 | 12017926 | 36539 | 263809 | 68934666 | 44.84 | 3447 |
| A5 | 104584 | 10128 | 959 | 455 | 161 | 57 | 69789205 | 34181027 | 17918519 | 14476454 | 9993245 | 6365792 | 29030 | 1017432 | 47071141 | 46.9 | 2352 |
| A6 | 225353 | 17903 | 2291 | 1196 | 496 | 240 | 1.56E+08 | 82230301 | 54907810 | 47309958 | 36537453 | 27580223 | 52703 | 643351 | 106011412 | 46.6 | 5983 |
| C1 | 98343 | 3436 | 435 | 254 | 115 | 52 | 45147101 | 15227377 | 9872231 | 8614708 | 6374160 | 4138022 | 12070 | 223748 | 20938811 | 46.52 | 3999 |
| C2 | 296009 | 34239 | 4043 | 1534 | 440 | 171 | 2.13E+08 | 1.18E+08 | 59571433 | 42601641 | 26207708 | 16939068 | 80635 | 413448 | 149666274 | 47.45 | 3071 |
| C3 | 213990 | 17001 | 1956 | 729 | 233 | 101 | 1.29E+08 | 57903965 | 30451375 | 22037209 | 14732246 | 10114084 | 50265 | 465627 | 80397210 | 46.67 | 2468 |
| C4 | 236316 | 33445 | 4952 | 2074 | 667 | 231 | 2.06E+08 | 1.3E+08 | 74756751 | 54885670 | 33750785 | 18744881 | 73668 | 256400 | 157689539 | 45.49 | 4363 |
| H1 | 54048 | 2425 | 509 | 265 | 100 | 31 | 29601071 | 13100986 | 9211868 | 7497917 | 4939881 | 2565761 | 7229 | 190750 | 16263329 | 43.76 | 7966 |
| H2 | 77531 | 6232 | 936 | 491 | 208 | 78 | 52642312 | 27681960 | 17889508 | 14829870 | 10425185 | 5906183 | 17105 | 184771 | 35027642 | 43.67 | 5416 |
| H3 | 54585 | 6298 | 1239 | 625 | 182 | 41 | 45652245 | 28957677 | 18862502 | 14579738 | 7854731 | 3053309 | 13450 | 128409 | 33870105 | 44.04 | 6941 |
| H4 | 295977 | 32225 | 3743 | 1595 | 457 | 157 | 2.05E+08 | 1.08E+08 | 55728560 | 40991685 | 23904163 | 13777034 | 82384 | 288985 | 142185469 | 48.32 | 2678 |
| H5 | 108953 | 5285 | 879 | 401 | 147 | 64 | 57424180 | 23402189 | 15002772 | 11705843 | 7899227 | 5054543 | 16860 | 205496 | 31071492 | 45.7 | 4509 |
| H6 | 61780 | 12851 | 1803 | 686 | 229 | 94 | 71108279 | 52276366 | 29683953 | 22083610 | 15283587 | 10499365 | 23716 | 469709 | 59881250 | 43.83 | 4915 |
